# Performance of Asphalt Mixtures Hybrid-Modified with Industrial Diatomite By-Products and Different Microfibres

**DOI:** 10.3390/polym18172151

**Published:** 2026-09-03

**Authors:** Mustafa Taha Aslan, Erol İskender, Cansu İskender, Osman Ünsal Bayrak, Turgay Ömür, Atakan Aksoy

**Affiliations:** 1 Graduate School of Natural and Applied Sciences, Karadeniz Technical University, Trabzon 61080, Türkiye; 2 Department of Civil Engineering, Faculty of Technology, Karadeniz Technical University, Trabzon 61830, Türkiye; eiskender@ktu.edu.tr; 3 Department of Civil Engineering, Avrasya University, Trabzon 61010, Türkiye; cansu.iskender@avrasya.edu.tr; 4 Department of Civil Engineering, Atatürk University, Erzurum 25240, Türkiye; unsalb@atauni.edu.tr; 5 Bentaş Bentonit Mining, Industry and Trade Limited Company, Ordu 52400, Türkiye; omur@bentasbentonit.com; 6 Department of Civil Engineering, Karadeniz Technical University, Trabzon 61080, Türkiye; aaksoy@ktu.edu.tr

**Keywords:** diatomite, fracture resistance, hybrid asphalt modification, microfibre reinforcement, moisture susceptibility, permanent deformation

## Abstract

This study evaluated asphalt mixtures incorporating two diatomite types (D1 and D2) at contents of 10% and 15% by binder weight and three fibre treatments added by aggregate weight: polyacrylonitrile (PAN) (0.065%), polyethylene terephthalate (PET) (0.10%), and basalt fibre (0.30%). An unmodified mixture and a mixture containing 5% styrene–butadiene–styrene (SBS) by binder weight were included as references. Performance was assessed using indirect tensile strength (ITS), tensile strength ratio (TSR), Cantabro loss, repeated load creep, and semi-circular bending (SCB) tests. Factorial analysis of variance indicated that diatomite type and content significantly affected ITS, diatomite content governed permanent deformation, and diatomite type was the principal factor affecting Cantabro loss. Significant interaction effects for the SCB parameters showed that fracture performance depended on the specific combination of diatomite and fibre treatments. Among the tested formulations, D1%10-PAN exhibited the highest ITS of 4712.36 kPa, representing numerical increases of 43.8% and 22.4% over the control and SBS-modified mixtures, respectively. D1%10-PET achieved the highest TSR (95.58%), whereas D2%10-PAN exhibited the lowest Cantabro loss (6.76%). Mixtures containing 15% diatomite generally showed lower permanent deformation, while D2%10-PAN achieved the highest flexibility index (14.003). The different performance trends observed for D1 and D2 are likely associated with differences in their physical characteristics, such as particle morphology and binder absorption behaviour, which may influence their interaction with the asphalt mastic and fibre reinforcement. No single formulation provided the best performance for every response. Overall, D2-based treatments improved resistance to particle loss, a higher diatomite content enhanced permanent deformation resistance, and 10% diatomite combined with PAN or PET treatment provided a more favourable balance of tensile, moisture, and fracture performance.

## 1. Introduction

Road transportation remains the dominant mode of passenger and freight transport worldwide, placing increasing demands on the structural capacity and long-term durability of asphalt pavements. Asphalt mixtures are multiphase composite materials consisting of bitumen, aggregates, and mineral filler, and their mechanical response is governed by viscoelastic behaviour that is strongly dependent on temperature and loading rate [1,2,3]. This temperature- and time-dependent response leads to different deformation and failure mechanisms under varying traffic and environmental conditions, making it difficult to maintain satisfactory pavement performance throughout the intended service life [4]. Pavement performance is also influenced by structural design parameters. For example, increasing asphalt layer thickness may enhance failure load and load-bearing capacity; however, it does not necessarily provide a corresponding improvement in permanent deformation or moisture resistance and may, under certain conditions, increase susceptibility to stripping [5]. Consequently, improvements in pavement performance require the combined consideration of structural design and material characteristics.

During service, asphalt pavements are exposed to repeated traffic loading, temperature fluctuations, and moisture infiltration, which contribute to the progressive development of low-temperature cracking, permanent deformation, and moisture-induced damage [6]. At low temperatures, thermal contraction generates tensile stresses within the pavement, while rapid temperature reductions reduce the stress relaxation capacity of asphalt mixtures, thereby promoting brittle behaviour and increasing the likelihood of early cracking [7]. At elevated temperatures, the viscous component of the asphalt response becomes more pronounced, resulting in the accumulation of permanent deformation under repeated wheel loading. Rutting is therefore governed by the combined effects of traffic loading, tyre–road interaction, contact pressure, temperature, and the mechanical properties of the mixture constituents [8,9]. Moisture represents another major deterioration mechanism. Water entering the pavement structure may increase pore-water pressure under repeated loading, weaken bitumen–aggregate adhesion, and promote stripping and subsequent material loss [10,11]. Because these distress mechanisms may occur concurrently, improving one performance characteristic without adversely affecting another remains a major challenge in asphalt mixture design.

Numerous additives have therefore been incorporated into bitumen and asphalt mixtures to improve their engineering performance and durability. These modifiers generally include polymers, mineral fillers, fibres, nanomaterials, and recycled or waste-derived materials [12]. Elastomeric and plastomeric polymers primarily improve elastic recovery, deformation resistance, and fatigue performance. Mineral fillers reinforce the asphalt mastic by modifying its stiffness and rheological response, whereas fibres may improve load transfer and restrict crack development through reinforcement and crack-bridging mechanisms. Nanomaterials and recycled materials can additionally improve binder–aggregate interaction, ageing resistance, and resource efficiency. Nevertheless, the existing literature has predominantly focused on basalt or lignin fibres and has generally examined a limited number of material combinations or performance indicators. A modifier that improves high-temperature stability may, for example, produce excessive stiffness and reduce low-temperature flexibility [12].

Polymer modification is among the most widely used methods for improving the rheological and mechanical properties of asphalt materials. (SBS) enhances the elastic recovery of the binder and generally improves rutting and fatigue resistance [13,14,15]. However, the wider use of SBS modification may be restricted by its relatively high cost, storage-stability problems, and susceptibility to ageing [16,17]. Mineral fillers and waste-derived additives have therefore received increasing attention as alternative or complementary modifiers. Although these materials can improve high-temperature performance by increasing the stiffness and viscosity of the asphalt mastic, excessive stiffening may promote brittle behaviour and adversely affect low-temperature cracking resistance [18]. Thus, the selection of an appropriate modifier requires a balance between deformation resistance and fracture performance.

Diatomite is a natural siliceous material that has attracted attention as a mineral modifier because of its porous structure, large specific surface area, and high binder-absorption capacity. These characteristics can increase the stiffness of the asphalt mastic and improve high-temperature stability and moisture resistance [19,20,21]. However, the stiffening effect of diatomite may reduce flexibility and increase the susceptibility of asphalt mixtures to brittle behaviour at low temperatures, particularly when excessive amounts are incorporated [22]. Its overall effectiveness may also depend on material-specific characteristics such as particle-size distribution, morphology, and composition. Therefore, diatomite type and content should be evaluated together rather than considering diatomite as a uniform modifier with a single performance response.

Fibre reinforcement offers a potential means of compensating for the reduction in flexibility associated with mineral modification. Fibres can form a reinforcing network within the asphalt mixture, improve stress transfer, restrict the development of microcracks, and delay crack propagation through crack-bridging mechanisms [23,24]. However, fibre performance depends strongly on material characteristics such as tensile strength, elastic modulus, surface properties, geometry, dispersion, and compatibility with the asphalt binder. PAN fibres have been investigated as micro-reinforcement materials for improving the mechanical performance of asphalt mixtures, particularly under cold-region conditions [25]. PET fibres have also been reported to improve the mechanical response of asphalt mixtures while offering a potentially cost-effective reinforcement alternative [26,27]. Basalt fibres, owing to their relatively high tensile strength, elastic modulus, and thermal stability, can contribute to tensile strength, load transfer, and resistance to cracking and environmental deterioration [28,29]. These distinct physical and mechanical characteristics suggest that different fibre treatments may interact differently with diatomite-modified asphalt mastics.

Previous studies have demonstrated the potential of combining diatomite with fibre reinforcement. Davar et al. [30] reported that the combined use of basalt fibre and diatomite powder enhanced the fatigue life and low-temperature tensile strength of hot-mix asphalt. Cheng et al. [31] similarly showed that a basalt fibre–diatomite compound modification system improved several performance characteristics of asphalt mixtures. Zhu et al. [32] further demonstrated that diatomite–basalt fibre composite modification could improve fatigue performance. In addition, Yue et al. [33] reviewed the performance of asphalt mixtures incorporating diatomite and lignin fibre and highlighted the potential of hybrid mineral–fibre modification. Collectively, these studies demonstrate that fibre reinforcement can complement the stiffening effect of diatomite. Nevertheless, the existing literature has predominantly focused on basalt or lignin fibres and has generally examined a limited number of material combinations or performance indicators.

A systematic comparison of different diatomite types combined with PAN, PET, and basalt fibre treatments across multiple pavement distress mechanisms remains limited. In particular, the effects of diatomite type, diatomite content, and fibre treatment on tensile response, moisture susceptibility, particle loss, permanent deformation, and fracture resistance have not been sufficiently evaluated within a common factorial framework. The identification of these effects is necessary because a hybrid system that performs well in terms of rutting resistance may not provide the same level of moisture or cracking resistance. Moreover, interactions among diatomite type, diatomite content, and fibre treatment may prevent the selection of a universally superior formulation.

Although previous studies have demonstrated that both diatomite and various fibre types can improve selected aspects of asphalt mixture performance [23,24,25,26,27,28,29,30,31,32,33], their findings are not entirely consistent. Reported improvements often vary depending on fibre type, diatomite characteristics, additive dosage, and the performance indicator considered [23,24,25,26,27,28,29,30,31,32,33]. Moreover, limited attention has been given to the interaction effects between different diatomite types and fibre treatments or to the associated performance trade-offs across multiple engineering properties [30,31,32,33]. These unresolved issues highlight the need for a systematic investigation using a comprehensive experimental and statistical framework. The novelty of this study lies in addressing an important research gap through the comprehensive evaluation of hybrid asphalt mixtures incorporating two different diatomite types together with three fibre treatments (PAN, PET, and basalt fibre). Unlike previous studies, which mainly focused on a single fibre type or limited performance indicators, this study simultaneously evaluates tensile strength, moisture susceptibility, particle loss, permanent deformation, and fracture resistance. Unlike previous investigations that primarily examined basalt fibre–diatomite systems [30,31,32,33], the present study systematically compares three different fibre treatments (PAN, PET, and basalt) combined with two industrial diatomite types, enabling a comprehensive evaluation of both the individual and interaction effects of the investigated factors across multiple pavement performance indicators. Furthermore, the study statistically quantifies the individual and interaction effects of diatomite type, diatomite content, and fibre treatment, providing a more comprehensive understanding of the performance trade-offs associated with hybrid modification systems. This approach advances the current understanding of hybrid asphalt modification beyond the evaluation of individual additive combinations by providing a systematic framework for identifying the governing factors, quantifying their interaction effects, and optimizing hybrid modification strategies according to targeted pavement performance requirements. In addition, the study contributes to the sustainable utilization of industrial diatomite by-products and recycled PET fibres, promoting the use of alternative materials with potential environmental and resource-efficiency benefits in asphalt pavement applications [12,26]. The experimental programme was based on a full factorial experimental design to systematically investigate the main and interaction effects of diatomite type, diatomite content, and fibre treatment on the performance of hybrid-modified asphalt mixtures. Accordingly, this study investigated the performance of hybrid-modified asphalt mixtures incorporating two diatomite types (D1 and D2), two diatomite contents (10% and 15% by binder weight), and three fibre treatments added by aggregate mass: PAN at 0.065%, PET at 0.10%, and basalt fibre at 0.30%. Because the fibres were incorporated at different material-specific dosages, they were evaluated as fibre treatments rather than as an isolated fibre-type factor. The factorial component of the experimental programme consisted of 12 diatomite–fibre combinations, while an unmodified mixture and a mixture containing 5% SBS by binder weight were included as reference mixtures. The mixtures were evaluated using indirect tensile strength, tensile strength ratio, Cantabro, repeated load creep, and semi-circular bending tests. The study aimed to determine how diatomite type, diatomite content, and fibre treatment affected the different performance responses, to identify statistically significant interactions among these factors, and to establish the performance trade-offs associated with the investigated hybrid modification systems.

## 2. Materials and Methods

### 2.1. Materials

**Figure 1:**
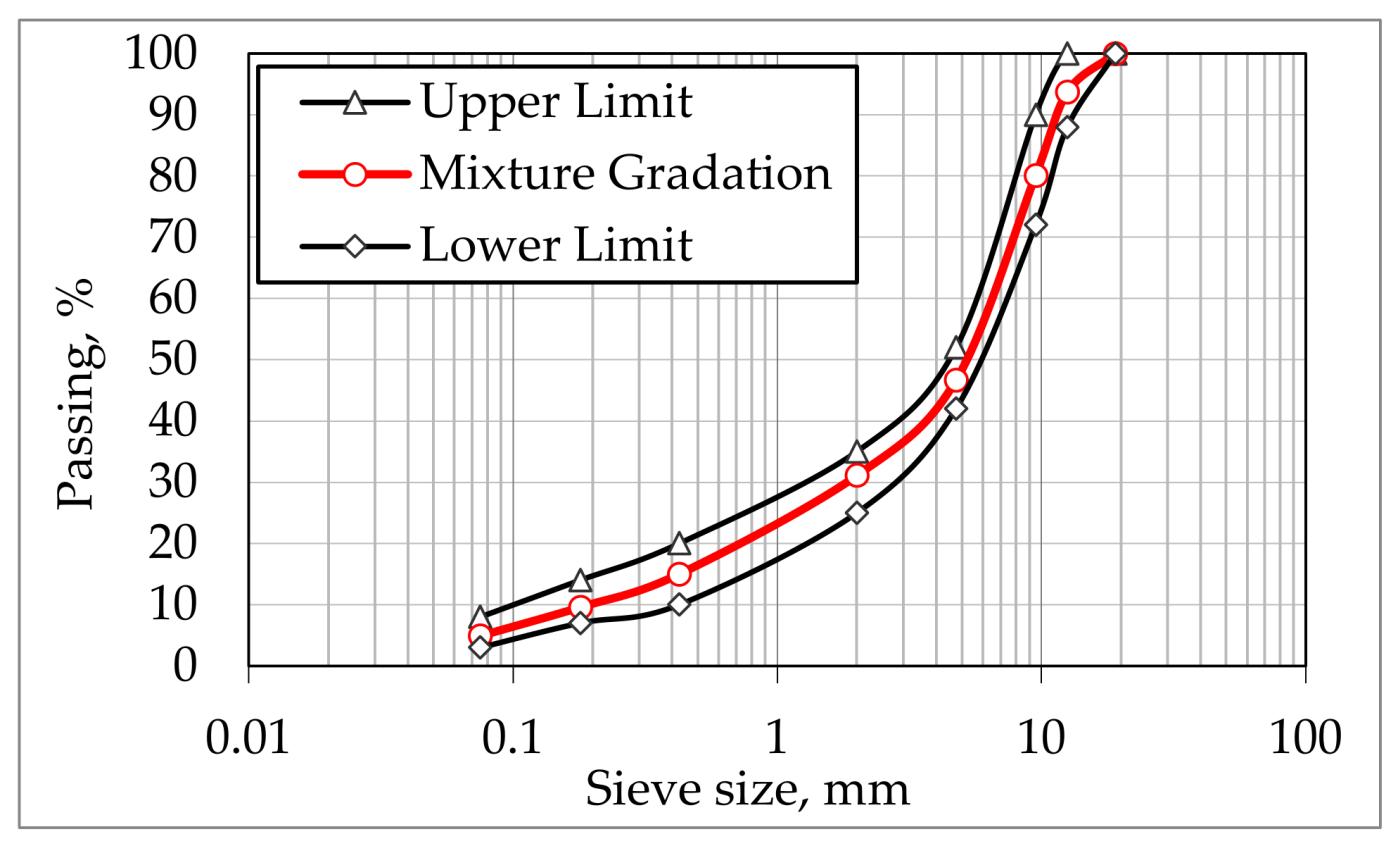
Aggregate gradation curve showing the combined aggregate gradation and the specified upper and lower gradation limits.

The study used 50–70 penetration grade asphalt cement, whose properties are shown in Table 1, and basalt aggregate in Table 2. According to the Turkish Highways Technical Specification [34], aggregate gradation suitable to produce dense-graded asphalt concrete was determined. Figure 1 presents the combined aggregate gradation together with the specified upper and lower gradation limits. The aggregate gradation was verified to ensure compliance with the specified gradation envelope prior to asphalt mixture preparation.

The aggregate properties presented in Table 2 and the supplementary aggregate characterization data provided in Appendix A (Table A1) were determined according to the following standards: filler, fine aggregate, and coarse aggregate specific gravity, apparent specific gravity, and water absorption in accordance with TS EN 1097-6 [40]; effective specific gravity of the mixture according to ASTM D-2041 [41]; polished stone value according to TS EN 1097-8 [42]; soundness according to TS EN 1367-2 [43]; Los Angeles abrasion loss according to AASHTO T-96 [44]; flakiness index according to BS 812-105.1 [45]; stripping resistance according to TS EN 12697-11 [46]; Micro-Deval abrasion loss according to TS EN 1097-1 [47]; and methylene blue value according to TS EN 933-9 [48].

**Table 2:** Aggregate Properties.

Properties	Test Result/Specification	Test Standard
Polished stone value	57.9/≥50 (PSV_50_)	TS EN 1097-8 [42]
Soundness (MgSO_4_) (%)	1.25/≤16 (MS_16_)	TS EN 1367-2 [43]
Los Angeles abrasion loss (%)	12/≤27 (LA_27_)	AASHTO T-96 [44]
Flakiness index (%)	11/≤25(FI_30_)	BS 812-105.1 [45]
Water absorption (%)	0.7/≤2.0 (WA_24_ 2.5)	TS EN 1097-6 [40]
Stripping resistance (%)	80/Without additive ≥60	TS EN 12697-11 [46]
Micro-Deval abrasion loss (%)	17/≤20 (M_DE_ 25)	TS EN 1097-1 [47]
Methylene Blue (%)	1/≤1.5 (MB1.5); ≤3.0 (MB3.0)	TS EN 933-9 [48]

**Figure 2:**
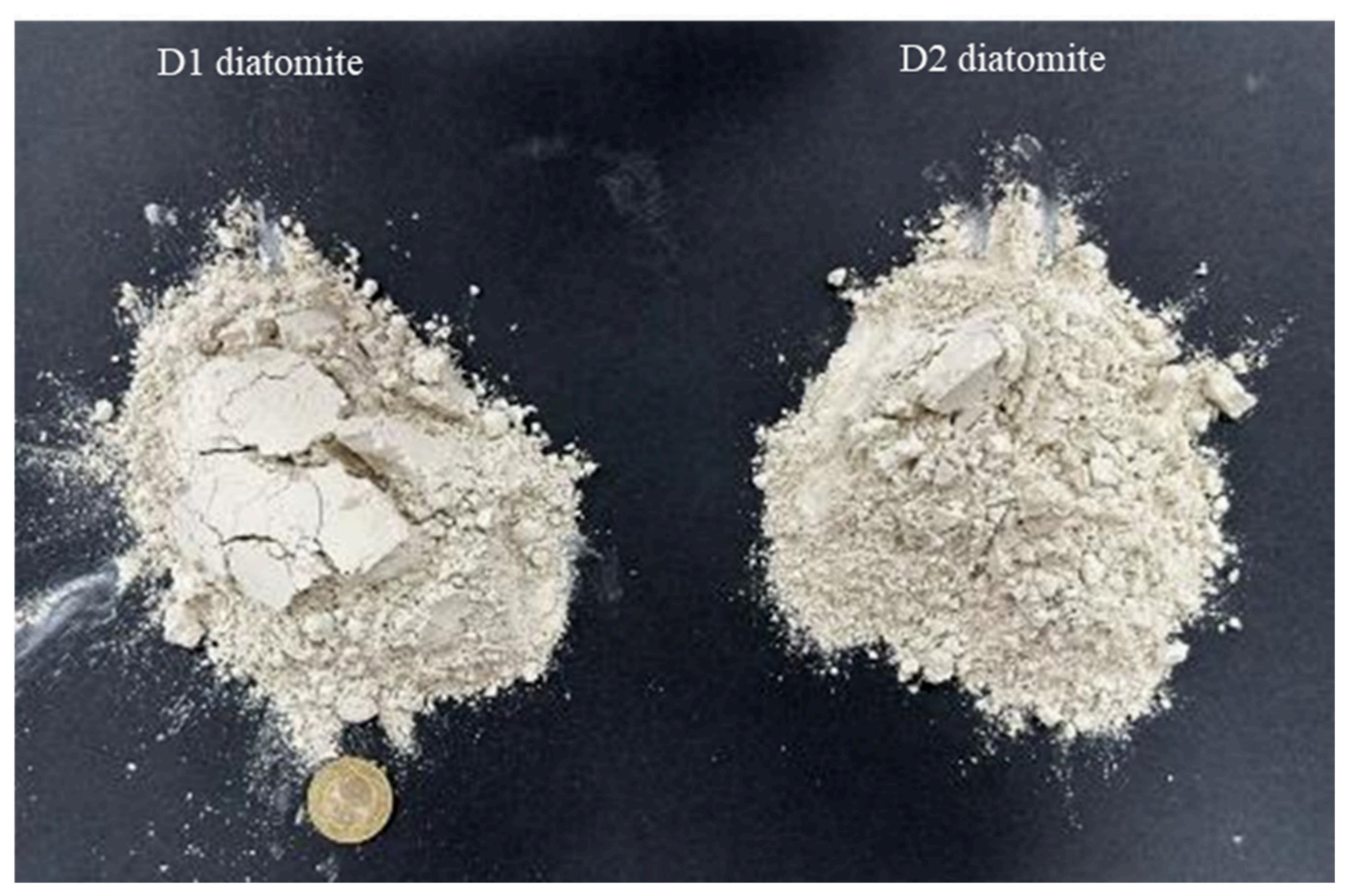
Photographs of the D1 and D2 diatomite types used as mineral modifiers in the asphalt mixtures.

**Figure 3:**
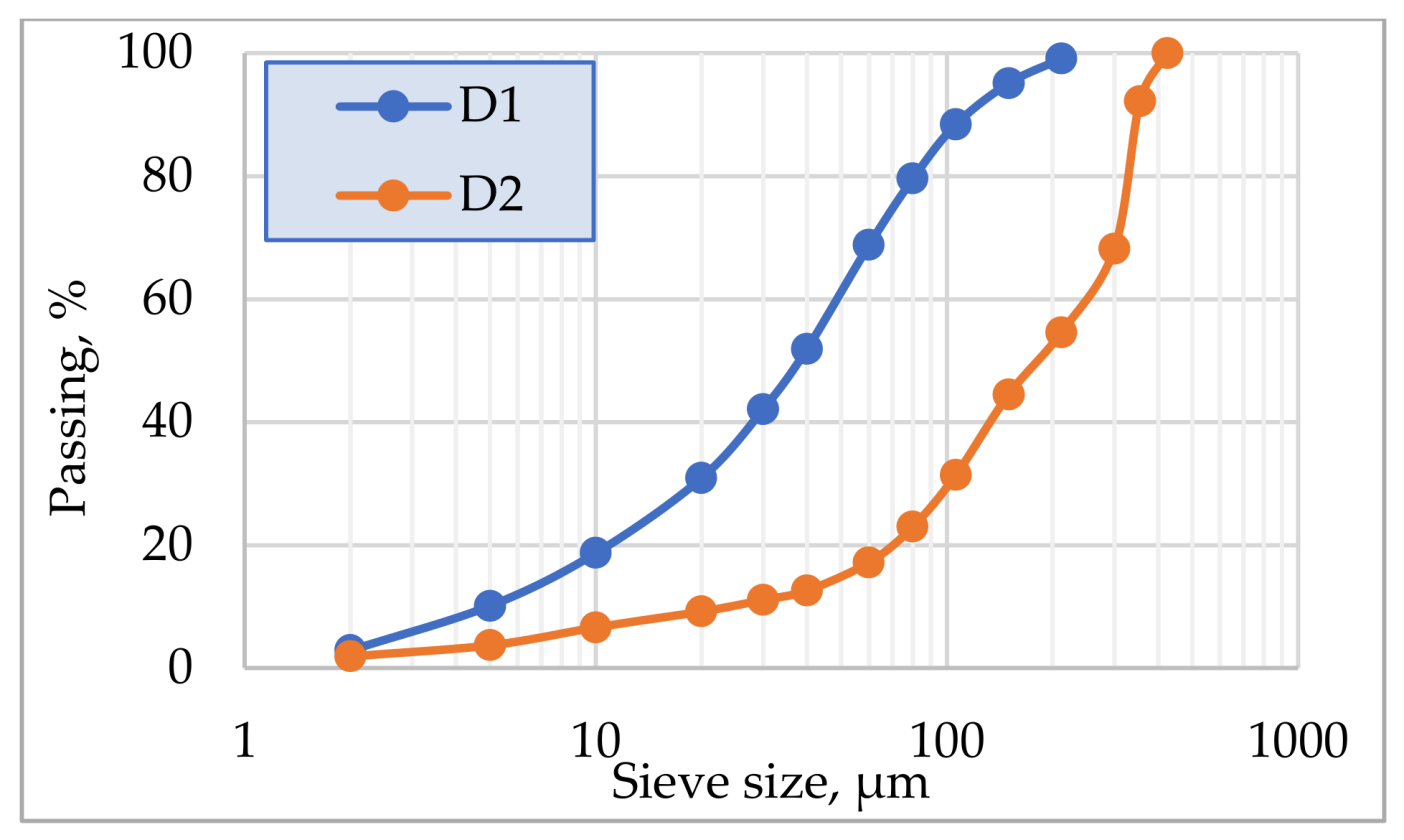
Particle size distribution curves of the two diatomites used in this study.

In this study, bitumen was modified with two different types of diatomite using the wet process at addition levels of 10% and 15% by weight of binder. The diatomite was obtained from Bentaş Bentonit A.Ş., Ordu, Türkiye as a fine by-product generated during the industrial processing of raw diatomite. After drying, grinding, and particle-size classification, the coarse fractions are commercially utilized for applications such as cat litter additives, pigments, bleaching, and filtration, whereas the fine fraction remaining below the screening size is currently underutilized. This study therefore investigates the potential use of this diatomite by-product as a sustainable mineral modifier in asphalt mixtures. Figure 2 shows the appearance of the diatomite, while Figure 3 presents the particle size distribution curves of both diatomite types.

Two commercially available diatomite (D1 and D2), supplied by Bentaş Bentonit A.Ş., were used as mineral fillers in this study. The maximum particle sizes of D1 and D2 were 212 µm and 425 µm, respectively. As shown by the particle size distribution curves, D1 exhibits a substantially finer particle size distribution than D2. Accordingly, the primary distinguishing material feature between D1 and D2 is their particle size distribution, with D1 providing a finer mineral structure than D2.

This difference is expected to influence the specific surface area available for binder interaction and, consequently, the performance of the hybrid-modified asphalt mixtures [19,20,49]. The comparison of two diatomite types with different particle size distributions was intended to evaluate how variations in diatomite characteristics influence the performance of hybrid-modified asphalt mixtures. This comparative approach provides scientific insight into the influence of diatomite physical characteristics on the effectiveness of hybrid modification systems and supports the selection of appropriate diatomite sources for different pavement performance requirements.

Diatomite is a natural mineral filler widely used in asphalt mixtures due to its porous microstructure, high silica content, and high bitumen absorption capacity [19,20,49]. The diatomite contents adopted in this study were selected based on optimum values reported in the literature. Du et al. [50] reported that the engineering performance of asphalt mixtures may deteriorate when the diatomite content exceeds 20%, with an optimum content of approximately 13%. Similarly, Zhang et al. [51] identified the optimum diatomite content to be between 10% and 13%, whereas Hou and Wu [52] demonstrated that incorporating 15% diatomite significantly improved rutting resistance. Based on these findings, diatomite contents of 10% and 15% by weight of binder were selected for this study.

**Figure 4:**
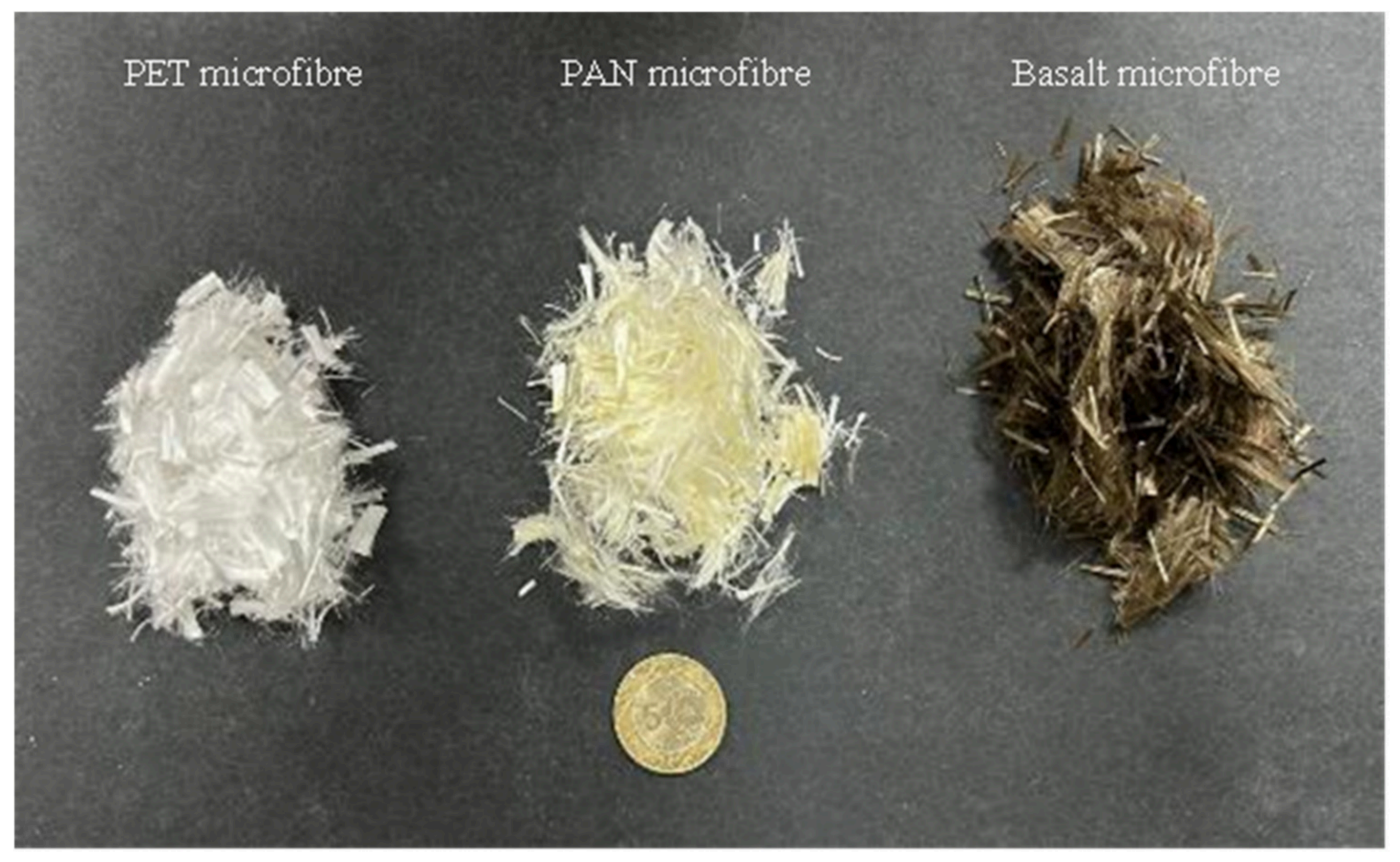
PET, PAN, and basalt microfibres used as reinforcing additives in the asphalt mixtures.

Modified binders were prepared using the wet process with a high-shear mixer at 180 °C, a mixing speed of 4500 rpm, and a mixing duration of 120 min. In addition to diatomite, PAN, PET, and basalt microfibres supplied by Pioneer Scientific Industry Co., Ltd. (Nanjing, China), were incorporated into the hybrid-modified asphalt mixtures. Figure 4 shows the microfibres used in this study.

PAN, PET, and basalt fibres were selected because they represent three reinforcement materials with distinctly different mechanical characteristics and sustainability attributes. PAN fibres were selected because of their high tensile strength, energy absorption capacity, and crack-bridging capability, whereas recycled PET fibres produced from waste plastics were selected as a cost-effective and environmentally sustainable reinforcement alternative. Basalt fibres were incorporated because of their high tensile strength, elastic modulus, and thermal stability, which contribute to improved mechanical performance. Although other fibre types, such as glass fibres, have also demonstrated promising performance in asphalt mixtures, their evaluation was beyond the scope of the present study. The selection of these fibre types was based on previous studies [24,25,26,27,28,29]. Although cellulose and polypropylene fibres have also been widely investigated for asphalt reinforcement [24], PAN and PET were selected in the present study because they provide distinctly different reinforcement mechanisms while enabling the evaluation of recycled PET as a sustainable reinforcement alternative. Consequently, the selected fibre treatments allowed a broader comparison of mechanical performance and sustainability within the proposed hybrid modification system.

According to the literature, although glass fibres possess a high elastic modulus, they exhibit relatively brittle behaviour, whereas the widespread application of carbon fibres remains limited owing to their high cost [25]. Slebi-Acevedo et al. [24] reported that PAN fibres improve rutting and skid resistance in asphalt mixtures. Similarly, PET fibres have been shown to enhance fatigue and rutting resistance while providing a cost-effective alternative [26,27]. In addition, the use of PET contributes to environmental sustainability through the recycling of waste plastics [26]. Liang et al. [29] demonstrated that basalt fibres improve tensile strength, dynamic stability, and stripping resistance, while also enhancing fatigue performance and providing economic benefits.

The mechanical properties of the microfibres are presented in Table 3. During asphalt mixture production, the fibres were incorporated using the dry process by first blending them with the heated aggregates. The fibre contents were calculated as a percentage of the total aggregate mass and added directly to the heated aggregates during mixing. The diatomite-modified binder was then added to the aggregate–fibre blend to produce the hybrid-modified asphalt mixtures. This dry mixing procedure was adopted to promote a more uniform distribution of the fibres throughout the aggregate skeleton before the addition of the modified binder. However, the quality of fibre dispersion was not quantitatively evaluated in the present study. Future investigations incorporating microstructural characterization techniques, such as SEM, are recommended to directly assess fibre dispersion and its influence on the performance of hybrid-modified asphalt mixtures.

The fibre contents were selected based on optimum values reported in the literature. Zhao et al. [28,53] reported that basalt fibres provide optimum mechanical performance at a content of approximately 0.3%, whereas Perca Callomamani et al. [25] and Perca et al. [54] identified optimum contents of 0.065% and 0.1% for PAN and PET fibres, respectively. Accordingly, basalt, PET, and PAN fibres were incorporated into the mixtures by the dry process at dosages of 0.3%, 0.1%, and 0.065% of the aggregate mass, respectively.

A 5% SBS dosage by weight of the base binder was selected as the benchmark modification level because it falls within the dosage range commonly used to produce conventional SBS-modified asphalt binders. At this concentration, SBS can form an effective polymer network and provide measurable improvements in the mechanical performance of asphalt mixtures. Therefore, the 5% SBS-modified mixture was included as a well-established reference system for evaluating the relative performance of the alternative modifiers investigated in this study [2,13].

A 5% SBS-modified binder was prepared as a reference for comparison with the hybrid-modified binders. The modifier used was Kraton D1192E (Kraton, Almere, The Netherlands), a linear styrene–butadiene–styrene thermoplastic elastomer supplied in granular form. According to the manufacturer’s technical data sheet [55], the polymer has a styrene/butadiene ratio of 29/71 and a specific gravity of 0.94, determined in accordance with ISO 2781 [56]. Its tensile strength, 300% modulus, and elongation at break, measured according to ISO 37 [57], are 33 MPa, 4.8 MPa, and 1000%, respectively. The polymer also has a Shore A hardness of 70 according to ASTM D2240 [58] and a melt flow rate below 1 g/10 min according to ISO 1133 [59]. Its linear molecular structure and high elongation capacity were expected to facilitate homogeneous dispersion and enhance the elastic response of the modified binder.

### 2.2. Methods

#### 2.2.1. Asphalt Mixture Design

The asphalt mixture design was conducted using 50/70 penetration-grade bitumen obtained from the İzmir Refinery. A multi-parameter experimental design was adopted to systematically evaluate the effects of diatomite type and content, together with fibre type, on the mechanical performance of asphalt mixtures. The experimental programme consisted of three main groups: unmodified (control), 5 wt.% SBS-modified, and diatomite-modified mixtures. In the diatomite-modified mixtures, two diatomite types with different particle size distributions, designated D1 and D2, were incorporated into the binder using the wet process at dosages of 10 wt.% and 15 wt.% by binder weight.

The mixture design was performed in accordance with the Marshall method [60,61] using a dense-graded aggregate gradation. For each binder group, 15 Marshall specimens (five binder contents with three replicate specimens each) were prepared to determine the optimum binder content in accordance with ASTM D6927 [60]. Specimens were compacted with 75 blows per face, and the binder content corresponding to an air void content of 4% was selected as the optimum binder content. The resulting mixture properties were verified to satisfy the relevant specification requirements. Because the fibre contents were relatively low, the optimum binder content determined separately for each binder group was retained for the corresponding fibre-treated mixtures. The optimum binder contents and corresponding mixture properties are presented in Table 4.

At the optimum binder contents determined from the Marshall mix design, control and diatomite–fibre hybrid-modified asphalt mixtures were prepared. A total of 12 hybrid-modified mixtures were produced by combining two diatomite types (D1 and D2) with three different microfibres (PAN, PET, and basalt) at the selected diatomite contents. For comparison, asphalt mixtures prepared with 5 wt.% SBS-modified binder and an unmodified (control) binder were also included in the experimental program and subjected to performance testing. The mixture designations and corresponding codes are presented in Table 5.

#### 2.2.2. Indirect Tensile Strength Test

The ITS test was conducted in accordance with TS EN 12697-23 [62] to determine the indirect tensile strength of asphalt mixtures and to assess their resistance to low-temperature cracking. The test determines the indirect tensile strength of cylindrical asphalt specimens by applying a compressive load along the vertical diameter, thereby inducing tensile stresses perpendicular to the loading direction. This method is widely used to evaluate the cracking resistance of asphalt mixtures [63,64]. Marshall specimens with a diameter of 101.6 mm were subjected to diametral compression at a constant loading rate of 50 mm/min. The peak load sustained by each specimen prior to failure was recorded, and the ITS values were calculated in accordance with TS EN 12697-23 [62]. The tests were performed at 0 °C. Before testing, all specimens were conditioned at this temperature for 24 h in a temperature-controlled chamber to ensure thermal equilibrium. For each mixture, three replicate specimens were tested, and the reported ITS values correspond to the mean of the three replicate measurements.

ITS was calculated using Equation (1):

ITS = (2000 × P)/(π × D × t)(1)

where

ITS: indirect tensile strength (kPa)

P: peak load (N)

D: specimen diameter (mm)

t: specimen thickness (mm).

The obtained ITS values were used to assess the tensile strength and resistance to low-temperature cracking of the asphalt mixtures.

#### 2.2.3. Tensile Strength Ratio Test

A moisture susceptibility test was conducted in accordance with the AASHTO T283-21 [65] standard to evaluate the resistance of asphalt mixtures to moisture-induced damage. This method is widely used to assess the susceptibility of asphalt mixtures to the loss of binder–aggregate adhesion caused by water. For each mixture, six Marshall specimens were prepared, of which three were assigned to the unconditioned (control) group and the remaining three to the conditioned group for moisture conditioning. The unconditioned specimens were immersed in a water bath at 25 °C for 2 h and then subjected directly to the ITS test.

The conditioned specimens were first subjected to vacuum saturation until a degree of saturation of 70–80% was achieved. The saturated specimens were then sealed in a watertight bag and frozen at −18 °C for 16 h. After freezing, the specimens were immersed in a water bath at 60 °C for 24 h and subsequently conditioned in a water bath at 25 °C for 2 h prior to the ITS test.

Following the conditioning procedures, all specimens were subjected to the ITS test. The indirect TSR was calculated from the measured ITS values using Equation (2).

(2) $$TSR=\frac{{ITS}_{conditioned}}{{ITS}_{unconditioned}}$$

The obtained TSR values were used to evaluate the moisture resistance of the asphalt mixtures. According to the Superpave volumetric mix design specification (AASHTO M 323) [66], asphalt mixtures tested in accordance with AASHTO T283-21 [65] should exhibit a minimum TSR of 0.80 to ensure adequate resistance to moisture-induced damage. This acceptance criterion has also been widely adopted in previous studies [67,68].

For each asphalt mixture, three conditioned and three unconditioned specimens were prepared and tested in accordance with AASHTO T283-21 [65]. The average ITS values obtained from each group were used to calculate the TSR.

#### 2.2.4. Cantabro Test

In order to determine the resistance of asphalt mixtures to particle loss, the Cantabro loss test was carried out using an adapted Cantabro procedure based on TS EN 12697-17 [69].

In the test, compacted asphalt specimens were placed individually in the Los Angeles abrasion tester and subjected to abrasion for 300 revolutions at a speed of 30–33 revolutions per minute. During the test, the specimens were rotated without the use of any abrasive steel balls. Prior to testing, three replicate Marshall specimens for each mixture were conditioned at 25 °C for at least 4 h to ensure uniform temperature distribution before the Cantabro test.

The sample weights were determined before and after the test, and the particle loss resulting from abrasion was calculated as a percentage. The Cantabro particle loss (CL) was determined using Equation (3):

(3) $$CL=100\times \frac{\left(W_{1}-W_{2}\right)}{W_{1}}$$

where

*CL*: Cantabro mass loss (%),

*W*_1_: specimen mass before the test (g),

*W*_2_: specimen mass after the test (g).

**Figure 5:**
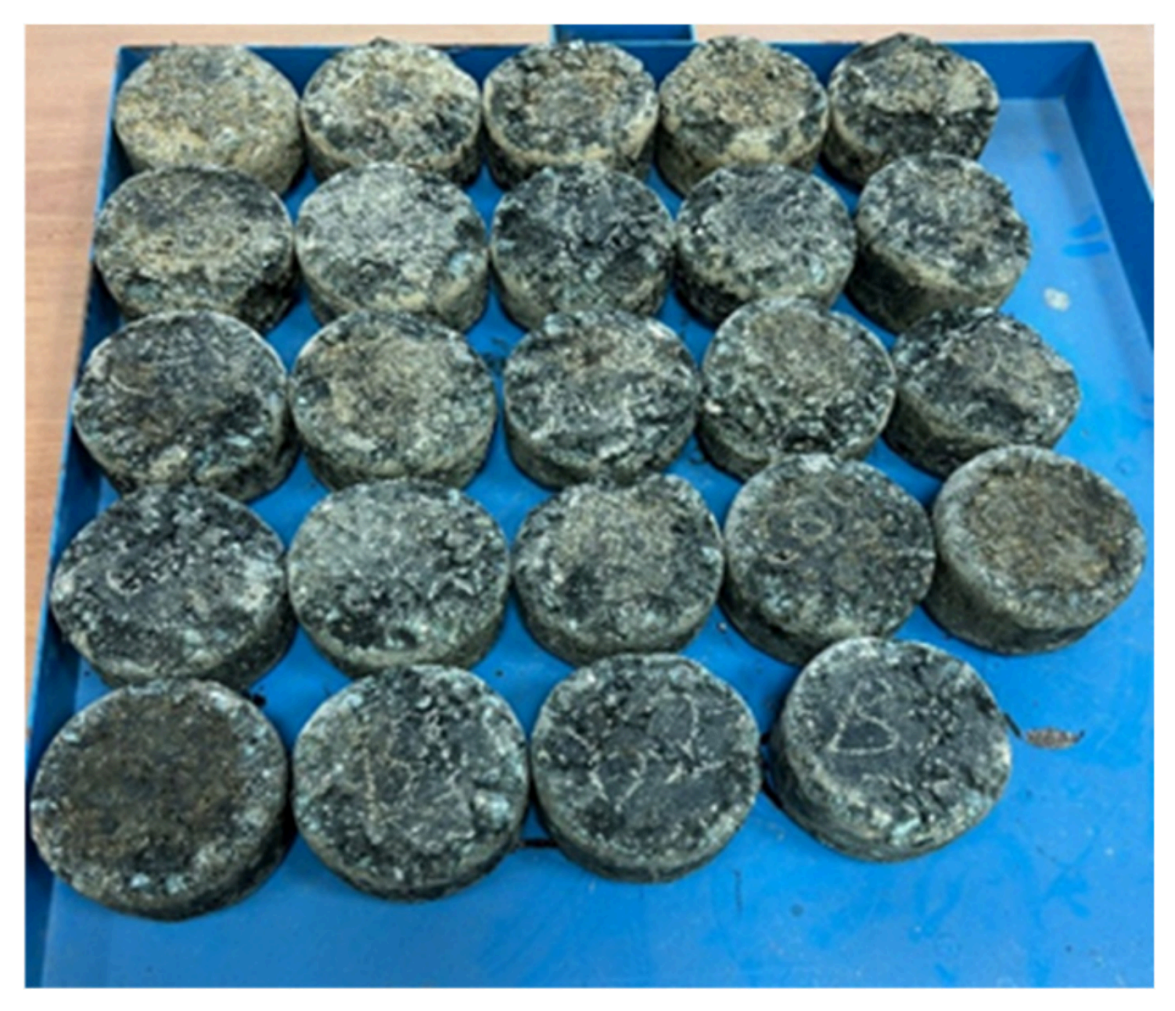
Representative asphalt mixture specimens after the Cantabro test.

The obtained results were used to evaluate the ravelling resistance of the asphalt mixtures. According to TS EN 12697-17 [69], a Cantabro mass loss of less than 20% indicates that the asphalt mixture possesses adequate resistance to ravelling. Although the Cantabro test was originally developed for Open-Graded Friction Courses (OGFC), for which maximum allowable mass losses of 20% for unaged specimens and 30% for aged specimens are specified ASTM D7064 [70], no generally accepted acceptance criterion has been established for Dense-Graded Asphalt (DGA) mixtures. Nevertheless, previous studies have suggested that DGA mixtures should exhibit considerably lower Cantabro loss values than OGFC mixtures because of their denser aggregate structure [67]. According to Doyle and Howard [71] and Cox et al. [72], Cantabro losses below approximately 15% are generally associated with satisfactory resistance to raveling and good mixture durability, whereas values exceeding 20% may indicate inadequate cohesion and reduced mechanical integrity. A schematic illustration of the Cantabro test procedure and representative specimens after testing are presented in Figure 5.

#### 2.2.5. Repeated Creep Test

A repeated creep test was conducted to evaluate the resistance of asphalt mixtures to permanent deformation and rutting. This test is widely used to characterize the deformation behaviour of asphalt mixtures under repeated loading and to assess their rutting resistance [73,74].

For each mixture, three replicate Marshall specimens were tested in accordance with TS EN 12697-25 [75] (Method A). The specimens were initially conditioned under an axial stress of 3 kPa for 2 min. Subsequently, the repeated creep test was conducted at 40 °C by applying a repeated axial stress of 95 kPa at a loading frequency of 0.5 Hz. A total of 10,000 loading cycles was applied to each specimen, and the accumulated permanent deformation was automatically recorded using computer software. The rutting resistance of the asphalt mixtures was evaluated using the cumulative permanent deformation and creep curves obtained from the tests. Furthermore, the viscoelastic behaviour of the mixtures was assessed by examining the development of permanent deformation with increasing loading cycles.

Tapkın et al. [76] recommended that repeated creep tests should be conducted at relatively low stress levels (generally not exceeding 206.9 kPa) and at temperatures not exceeding 40 °C. Higher stress levels or temperatures may cause premature specimen failure before the completion of the test.

**Figure 6:**
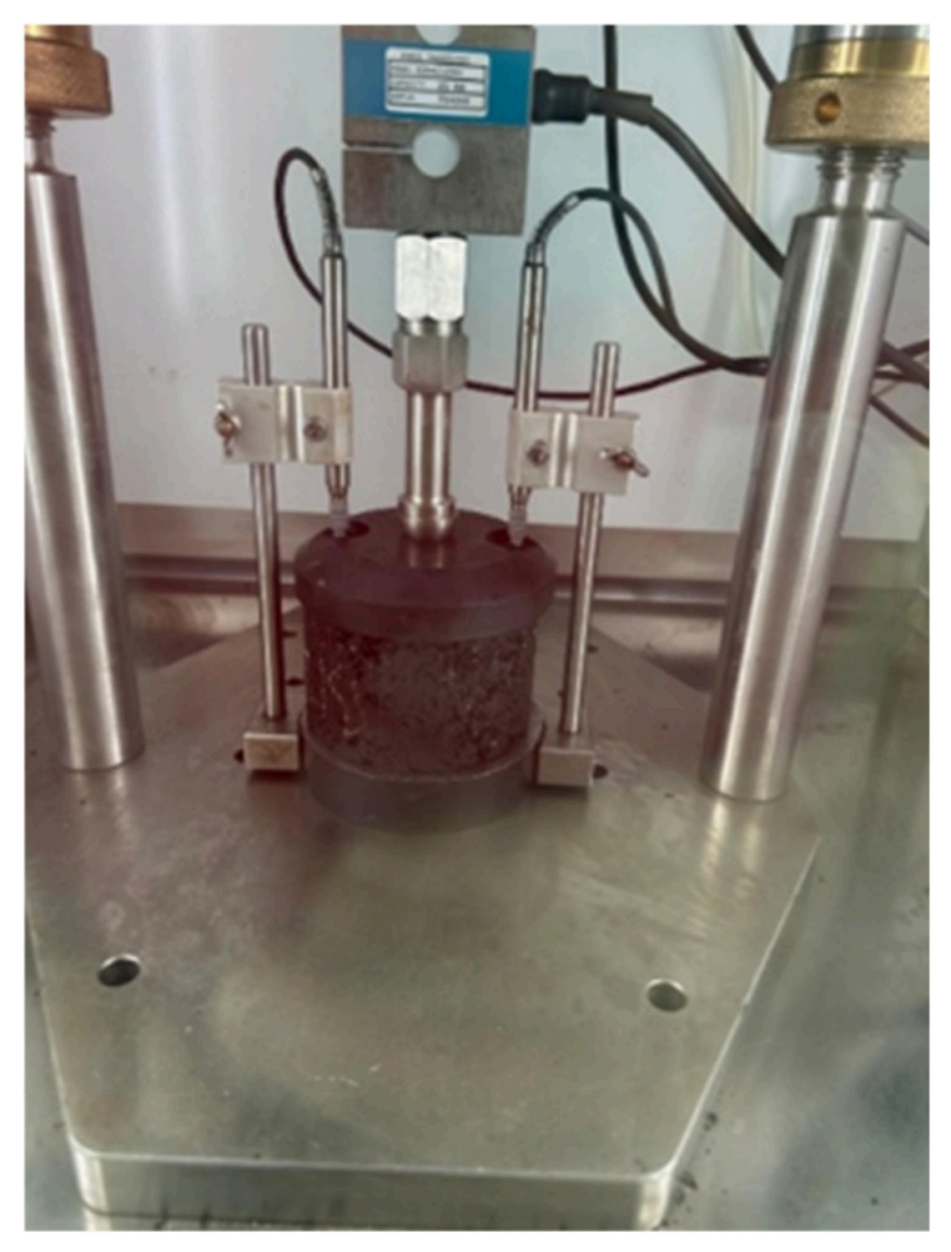
Experimental setup for the repeated creep test used to evaluate the permanent deformation behaviour of asphalt mixtures.

A schematic illustration of the repeated creep test setup is shown in Figure 6.

#### 2.2.6. Intermediate Temperature Cracking Resistance (SCB Test)

The mechanical behaviour of hot-mix asphalt (HMA) depends on temperature and loading rate. The linear elastic fracture mechanics (LEFM) approach is generally applicable at low temperatures, whereas the elastic-plastic fracture mechanics (EPFM) approach is more appropriate at intermediate and high temperatures [77].

To evaluate the cracking resistance of asphalt mixtures at intermediate temperatures, the SCB test was conducted adapted SCB procedure. The SCB test evaluates crack initiation and propagation under bending loads applied to a notched semi-circular specimen and provides fracture parameters such as fracture energy (G_f_) and the flexibility index (FI) [78,79,80].

The SCB specimens were prepared by cutting Marshall-compacted cylindrical specimens into semi-circular halves. For each mixture, three Marshall specimens were prepared and subsequently cut into two equal halves, resulting in a total of six semi-circular SCB specimens. A notch with a depth of 15 ± 1 mm was introduced at the centre of each specimen. The specimen geometry and air void content complied with the requirements of AASHTO TP 124-18 [81].

The tests were conducted at 25 °C under displacement-controlled loading at a constant loading rate of 50.8 mm/min, following the loading protocol of AASHTO TP 124-18 [81]. Although this standard was originally established for 150 mm diameter SCB specimens, the same loading procedure was adopted for the 100 mm Marshall-compacted specimens used in this study to ensure consistency with the standardized test protocol.

The tests were conducted at a temperature of 25 °C under displacement-controlled loading at a constant loading rate of 50.8 mm/min. Monotonic loading was applied throughout the test, and the load–load line displacement (P–LLD) data were recorded continuously. The fracture work (W_f_) was determined by numerically integrating the area under the load–LLD curve in accordance with AASHTO TP 124-18 [81].

The Gf was calculated by dividing the fracture work by the ligament area, as expressed in Equations (4) and (5) [81,82]:

(4) $$A_{lig}=Ligament\;area\;({\mathrm{mm}}^{2})=\left(r-a\right)\times t$$

(5) $$G_{f}=Fracture\;energy\;(\frac{J}{m^{2}})=\frac{W_{f}}{A_{lig}}\times 10^{6}$$

where

*r*: Specimen radius (mm),

*a*: Notch length (mm),

*t*: Specimen thickness (mm).

*W_f_* is expressed in kN·mm (=J), *A_lig_* mm^2^, and *G_f_* in J/m^2^.

The FI was calculated by dividing the fracture energy by the post-peak slope of the P–LLD curve, as expressed in Equation (6) [78,79]. For each mixture, the reported fracture parameters were obtained from six SCB specimens, and the experimental results were analysed using the arithmetic mean and standard deviation to evaluate the repeatability and reliability of the measurements.

(6) $$FI=\frac{G_{f}}{\left|m\right|}\times A$$

**Figure 7:**
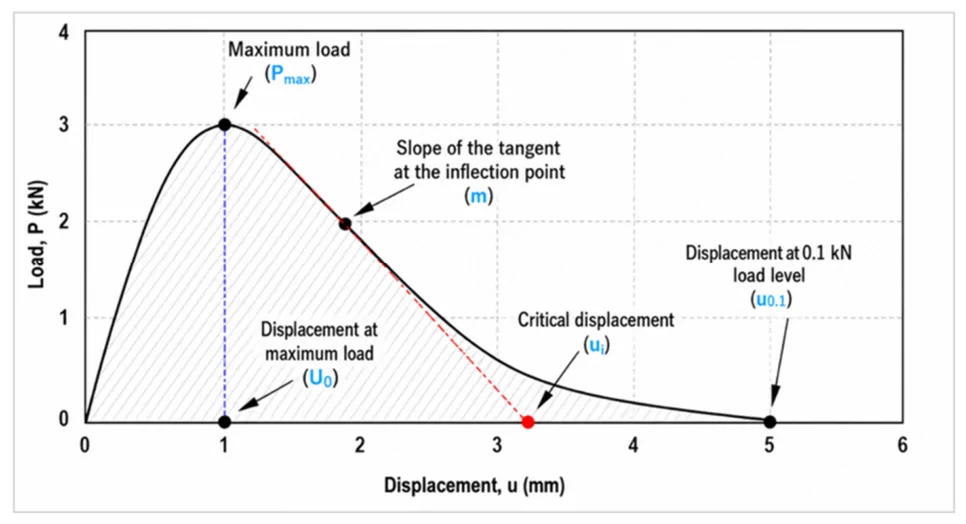
Schematic load–displacement curve for the SCB test (adapted from AASHTO TP 124-18, [81]; Kasil and Çodur, [83]).

**Figure 8:**
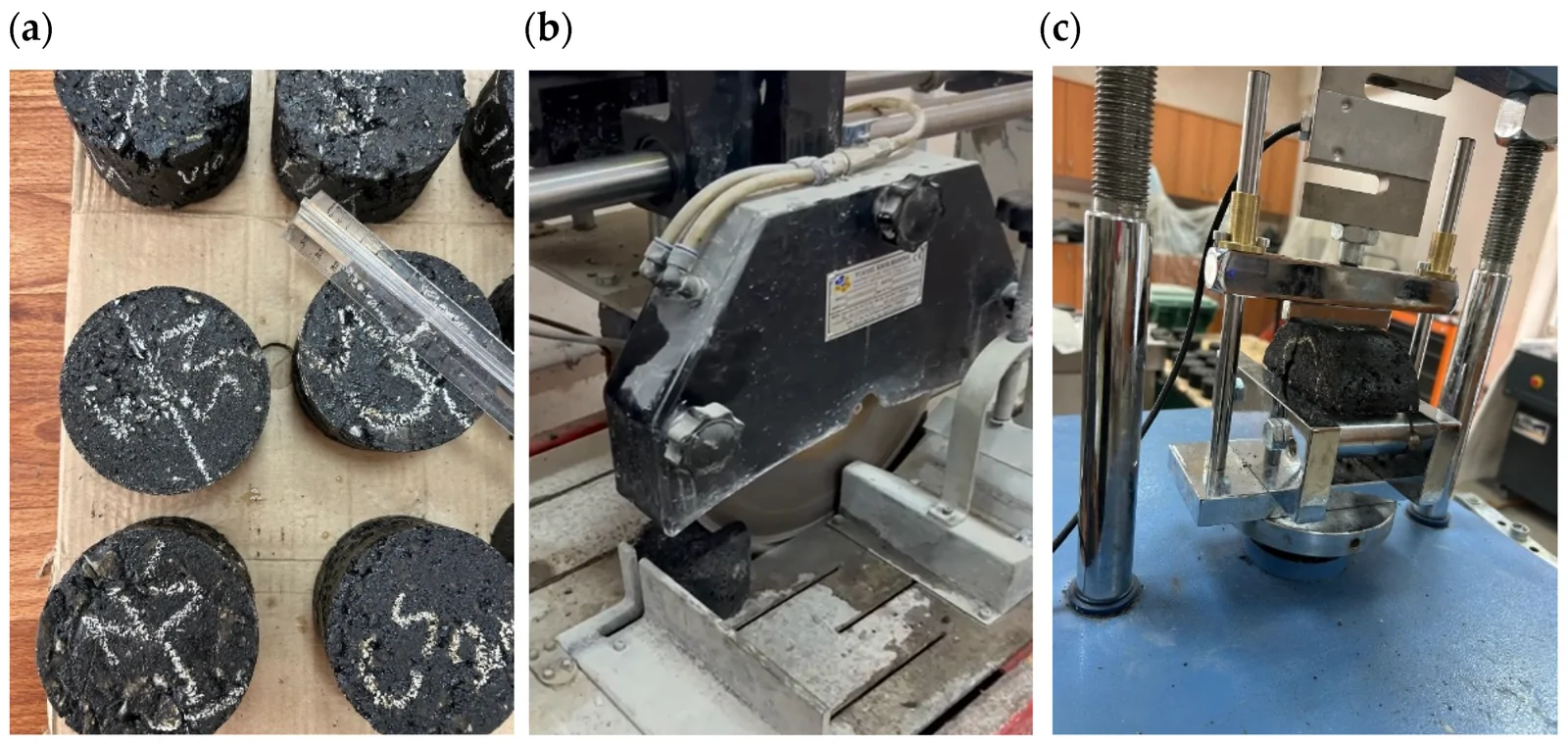
Images from the SCB test: (**a**) specimen preparation, (**b**) specimen cutting, and (**c**) the SCB test setup.

The coefficient *A* was set to 0.01, while *m* denotes the slope of the tangent at the inflection point on the descending branch of the load–displacement curve. The crack resistance of the asphalt mixtures was evaluated based on the obtained *G_f_* and *FI* values. The schematic load–displacement curve and the stages of the SCB test are illustrated in Figure 7 and Figure 8, respectively.

#### 2.2.7. Statistical Analysis

The experimental results are presented as mean values with their corresponding standard deviations. A 2 × 2 × 3 full factorial experimental design was employed to evaluate the effects of diatomite type, diatomite content, and fibre type on the performance of diatomite–fibre hybrid asphalt mixtures. In the statistical model, diatomite type (D1 and D2), diatomite content (10% and 15%), and fibre treatment: PAN at 0.065%, PET at 0.10%, and basalt fibre at 0.30%.

A three-way analysis of variance (ANOVA) was performed on the indirect tensile strength, Cantabro particle loss, permanent deformation after 10,000 loading cycles, obtained from the SCB test, the absolute value of the post-peak slope (∣m∣), and the FI. The analysis evaluated the main effects of diatomite type, diatomite content, and fibre type, as well as their two-way and three-way interaction effects. When interaction effects were not statistically significant, the main effects were interpreted directly. Conversely, when significant interaction effects were detected, the corresponding combinations of factor levels were considered in the interpretation of the results.

As the untreated control and the 5% SBS-modified mixtures were not included in the factorial experimental design, they were excluded from the factorial ANOVA model and compared descriptively with the diatomite–fibre hybrid mixtures. For the evaluation of moisture susceptibility, the TSR values were reported descriptively, whereas the conditioned and unconditioned ITS results were analysed using a two-way ANOVA, with mixture type and conditioning status as fixed factors, together with their interaction.

The assumptions of ANOVA were assessed based on the model residuals. When violations of the assumptions were detected, statistical inferences were based on the HC3 heteroscedasticity-consistent covariance estimator. Statistical significance was determined at a significance level of *p* < 0.05. All statistical analyses were performed using IBM SPSS Statistics 30.0.

## 3. Results and Discussion

### 3.1. Indirect Tensile Strength Test

**Figure 9:**
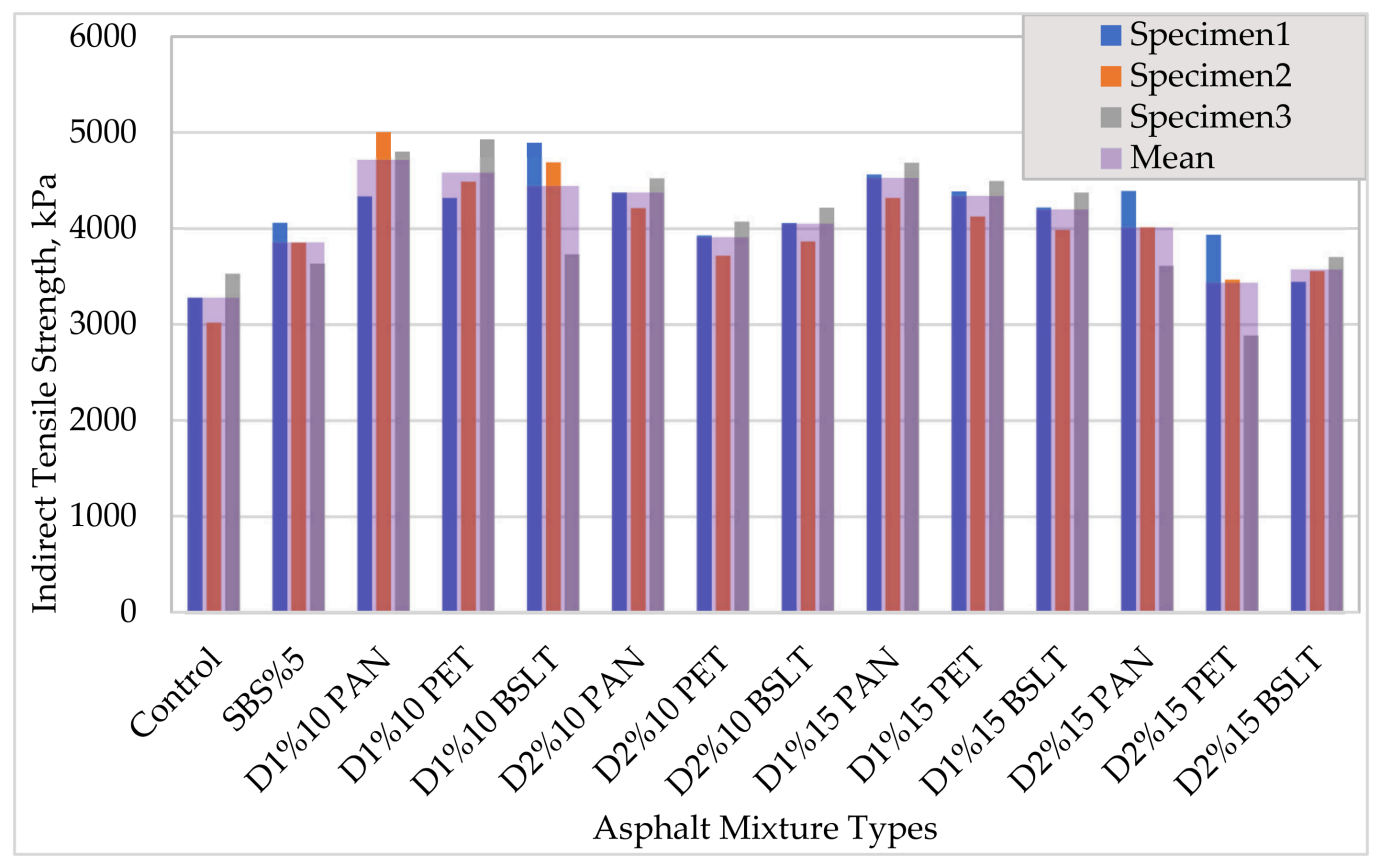
ITS values of asphalt mixtures with different hybrid modification systems.

The ITS tests were performed at 0 °C on three replicate specimens to evaluate the low-temperature performance of the asphalt mixtures. The corresponding results are presented in Figure 9.

The results of the ITS tests indicate that the tensile strength of the asphalt mixtures was jointly influenced by the diatomite type, diatomite content, fibre type, and the interactions among these factors. Conducting the ITS test at 0 °C enabled the low-temperature tensile behaviour and crack initiation resistance of the mixtures to be evaluated. Therefore, the ITS test serves as an important mechanical performance indicator of the ability of asphalt mixtures to resist tensile stresses and maintain their structural integrity under low-temperature conditions.

The average ITS of the untreated control mixture was 3276.34 kPa, whereas the mixture modified with 5% SBS exhibited an average ITS of 3850.00 kPa, corresponding to an increase of approximately 17.5%. This improvement may be associated with the elastomeric nature of SBS, which enhances the elastic recovery of the binder, promotes a more uniform stress distribution, and delays crack initiation. Previous studies have similarly reported that SBS modification improves the tensile, deformation, and fatigue performance of asphalt mixtures by increasing the elasticity and toughness of the asphalt binder [16,84]. However, as shown in Figure 9, several diatomite–fibre hybrid mixtures exhibited higher ITS values than the 5% SBS-modified mixture. This finding suggests that the combined effect of diatomite and fibre reinforcement can provide greater improvement in tensile strength than polymer modification alone, highlighting the effectiveness of the proposed hybrid reinforcement system.

The highest ITS value was obtained for the D1%10 PAN mixture, with an average value of 4712.36 kPa. This value was approximately 43.8% higher than that of the untreated control mixture and 22.4% higher than that of the 5% SBS-modified mixture. The D1%10 PET and D1%10 BSLT mixtures also exhibited high ITS values of 4577.78 and 4439.05 kPa, respectively. These findings indicate that D1-type diatomite, particularly at a content of 10%, formed an effective mastic–fibre reinforcement system when combined with PAN, PET, or basalt fibres. Owing to its high porosity and specific surface area, diatomite can increase the stiffness and load-bearing capacity of the asphalt mastic, whereas fibres are generally reported to improve ductility and may contribute to crack resistance, possibly through crack-bridging mechanisms reported in previous studies [19,20,23,85]. The combined effects of mastic stiffening and fibre reinforcement may therefore explain the high ITS values obtained for the D1%10 mixtures.

The effect of diatomite on asphalt mixture performance has generally been attributed to its porous structure and high specific surface area reported in the literature, which may increase bitumen absorption and contribute to increased asphalt mastic stiffness. It is generally considered that diatomite may absorb part of the binder, thereby influencing the viscosity of the asphalt mastic. However, the accompanying increase in stiffness may also increase the susceptibility of the mixture to brittle behaviour at low temperatures, particularly as the diatomite content increases [19,20,49]. Consequently, the influence of diatomite content is not linear. When the optimum dosage is exceeded, the effective bitumen film thickness may decrease, resulting in excessive mastic stiffness and earlier crack initiation under tensile loading.

As shown in Figure 9, increasing the D1 diatomite content from 10% to 15% generally resulted in a slight reduction in ITS, although relatively high strength values were still maintained. For example, the average ITS of the D1%10-PAN mixture decreased from 4712.36 to 4522.67 kPa when the diatomite content was increased to 15%. Similarly, the ITS of the D1%10-PET mixture decreased from 4577.78 to 4335.78 kPa in the corresponding D1%15-PET mixture. These results suggest that a diatomite content of 10% provides a more favourable balance for maximizing the ITS of mixtures containing D1-type diatomite. Although increasing the diatomite content to 15% further reinforced the asphalt mastic, it may also have reduced the effective bitumen film thickness and adversely affected workability and fibre–binder adhesion. Since mineral fillers enhance the stiffness of the asphalt mastic, excessive stiffening can increase the susceptibility of asphalt mixtures to brittle behaviour under low-temperature conditions. Therefore, the slight reduction in ITS observed for the D1%15 mixtures is consistent with the mechanisms reported in previous studies [86,87].

A more pronounced reduction in ITS was observed for the D2 mixtures as the diatomite content increased from 10% to 15%. Although the D2%10 PAN mixture exhibited a relatively high ITS value of 4370.04 kPa, this value decreased to 4005.23 kPa for the corresponding D2%15 PAN mixture. Likewise, the D2%15 PET and D2%15 BSLT mixtures exhibited average ITS values of 3429.78 and 3568.19 kPa, respectively, both of which were lower than that of the 5% SBS-modified mixture. These findings indicate that D2-type diatomite was not equally compatible with all fibre types, particularly at the higher diatomite content of 15%. One possible explanation is that the physical characteristics of D2-type diatomite, including its relatively coarse particle size and porous structure, may influence the binder–mastic balance at higher addition levels. This may increase binder absorption and reduce the effective bitumen film thickness surrounding the aggregate particles and fibres, potentially limiting the reinforcing contribution of the fibres and resulting in lower tensile strength. The performance differences observed between the D1 and D2 based mixtures are therefore interpreted primarily in relation to their different particle size distributions. Mineralogical characterization of the two diatomite types was beyond the scope of the present study; consequently, no conclusions can be drawn regarding the influence of mineralogical composition on the observed behaviour. However, this interpretation remains speculative because the present study did not include microstructural characterization. Nevertheless, the observed trend is consistent with previous studies reporting that excessive mineral filler contents can adversely affect the balance between mastic stiffness and binder availability, thereby reducing the low-temperature tensile performance of asphalt mixtures [88].

When the mixtures were evaluated according to fibre type, the overall performance generally followed the order PAN > PET > basalt fibre. Mixtures reinforced with PAN fibres consistently exhibited the highest or among the highest ITS values, regardless of the diatomite type or content. This finding suggests that PAN fibres provided more effective reinforcement by improving stress distribution within the asphalt mastic and delaying the initiation and propagation of microcracks. Previous studies have reported that PAN fibres enhance the rutting resistance and interfacial bonding of asphalt mixtures, whereas PET fibres provide a cost-effective and sustainable alternative that improves fatigue resistance and rutting performance [24,26,27]. Accordingly, the superior ITS values obtained for the D1%10 PAN and D1%15 PAN mixtures indicate that PAN fibres acted as an effective micro-reinforcement, enabling the mixtures to better resist tensile stresses under low-temperature conditions.

PET fibre-reinforced mixtures also exhibited high performance, particularly those incorporating D1-type diatomite. The D1%10 PET mixture achieved an average ITS value of 4577.78 kPa, indicating that PET fibres can effectively enhance tensile strength when combined with an appropriate diatomite type and optimum additive content. However, the ITS value decreased to 3429.78 kPa for the D2%15 PET mixture, suggesting that PET fibres may not provide optimum compatibility with D2-type diatomite at higher addition levels. This reduction may be attributed to excessive binder absorption by the diatomite, which can reduce the effective bitumen film thickness and consequently weaken fibre–binder interaction.

In contrast, the performance of basalt fibre-reinforced mixtures was more variable. Although the D1%10 BSLT mixture exhibited a relatively high average ITS value, the larger error bars indicate greater variability among replicate specimens. Basalt fibres possess a high elastic modulus and tensile strength and therefore have the potential to improve the mechanical performance of asphalt mixtures [28,29]. However, their relatively high stiffness and density may hinder uniform dispersion throughout the asphalt mixture. In addition, fibre agglomeration or inadequate fibre–binder bonding can reduce the efficiency of the crack-bridging mechanism, resulting in greater variability in mechanical performance. Wu et al. [89] reported that the effectiveness of basalt fibres in improving crack resistance is strongly influenced by fibre distribution and the quality of the fibre–binder interface. Therefore, although basalt fibres exhibit considerable reinforcement potential, achieving consistent performance requires more precise control of mixture preparation and fibre dispersion than is necessary for PAN or PET fibres.

The relatively low ITS values obtained for the D2%15 PET and D2%15 BSLT mixtures indicate that increasing the diatomite content beyond the optimum level may disrupt the balance among bitumen absorption, mastic stiffness, fibre dispersion, and fibre–binder interaction. As a result, the beneficial effects of fibre reinforcement may be diminished, leading to a reduction in low-temperature tensile performance. This observation is consistent with previous studies reporting that, although diatomite improves the high-temperature stability and stiffness of asphalt mixtures up to an optimum content, excessive addition can adversely affect low-temperature performance by reducing the effective bitumen film thickness and increasing the brittleness of the mixture [19,33,51].

Among all mixtures evaluated, the D1%10 PAN mixture exhibited the highest ITS value, indicating that this combination provided the most effective reinforcement under low-temperature conditions. In this mixture, the stiffening effect of D1-type diatomite on the asphalt mastic was effectively complemented by the crack-bridging capability of PAN fibres, resulting in improved stress distribution and delayed microcrack initiation. Consequently, the D1%10 PAN mixture achieved substantially higher tensile strength than both the untreated control and the 5% SBS-modified mixture. The D1%10 PET and D1%10 BSLT mixtures also demonstrated favourable ITS performance. In contrast, the comparatively low ITS values of the D2%15 PET and D2%15 BSLT mixtures suggest that high contents of D2-type diatomite, when combined with certain fibre types, may not provide an optimum balance between stiffness and ductility. Overall, these findings demonstrate that the performance of diatomite–fibre hybrid-modified asphalt mixtures depend not only on the amount of filler added but also on the appropriate combination of diatomite type, filler content, and fibre type.

Although microstructural characterization (e.g., SEM) was beyond the scope of the present study, the observed increase in ITS is consistent with the improved aggregate–binder interaction and crack-bridging mechanisms reported for diatomite- and fibre-modified asphalt mixtures in previous studies [23,24,30,31,32,33]. Nevertheless, future investigations incorporating fracture surface observations and microstructural characterization are recommended to provide direct evidence of these reinforcement mechanisms.

An increase in indirect tensile strength (ITS) indicates an improved ability of the asphalt mixture to resist tensile stresses induced by traffic loading and environmental effects. Higher ITS values generally reflect stronger aggregate–binder adhesion and improved internal cohesion, thereby reducing the likelihood of crack initiation and propagation. Consequently, mixtures with higher ITS are expected to exhibit enhanced structural integrity, greater resistance to fatigue cracking, and improved long-term pavement durability.

### 3.2. Evaluation of Moisture Susceptibility

**Figure 10:**
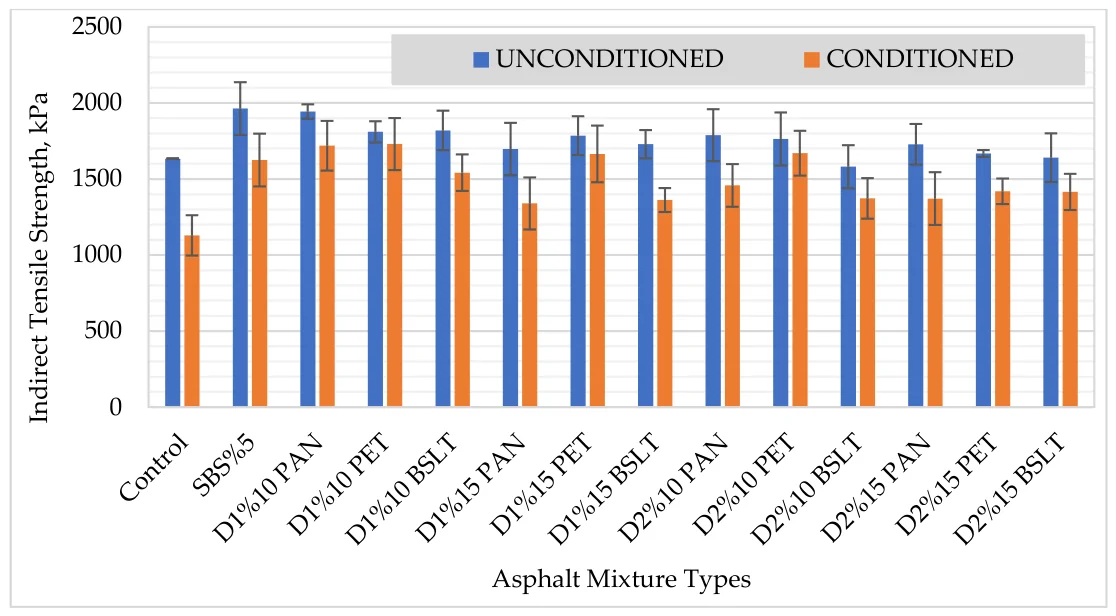
ITS values of conditioned and unconditioned asphalt mixtures.

**Figure 11:**
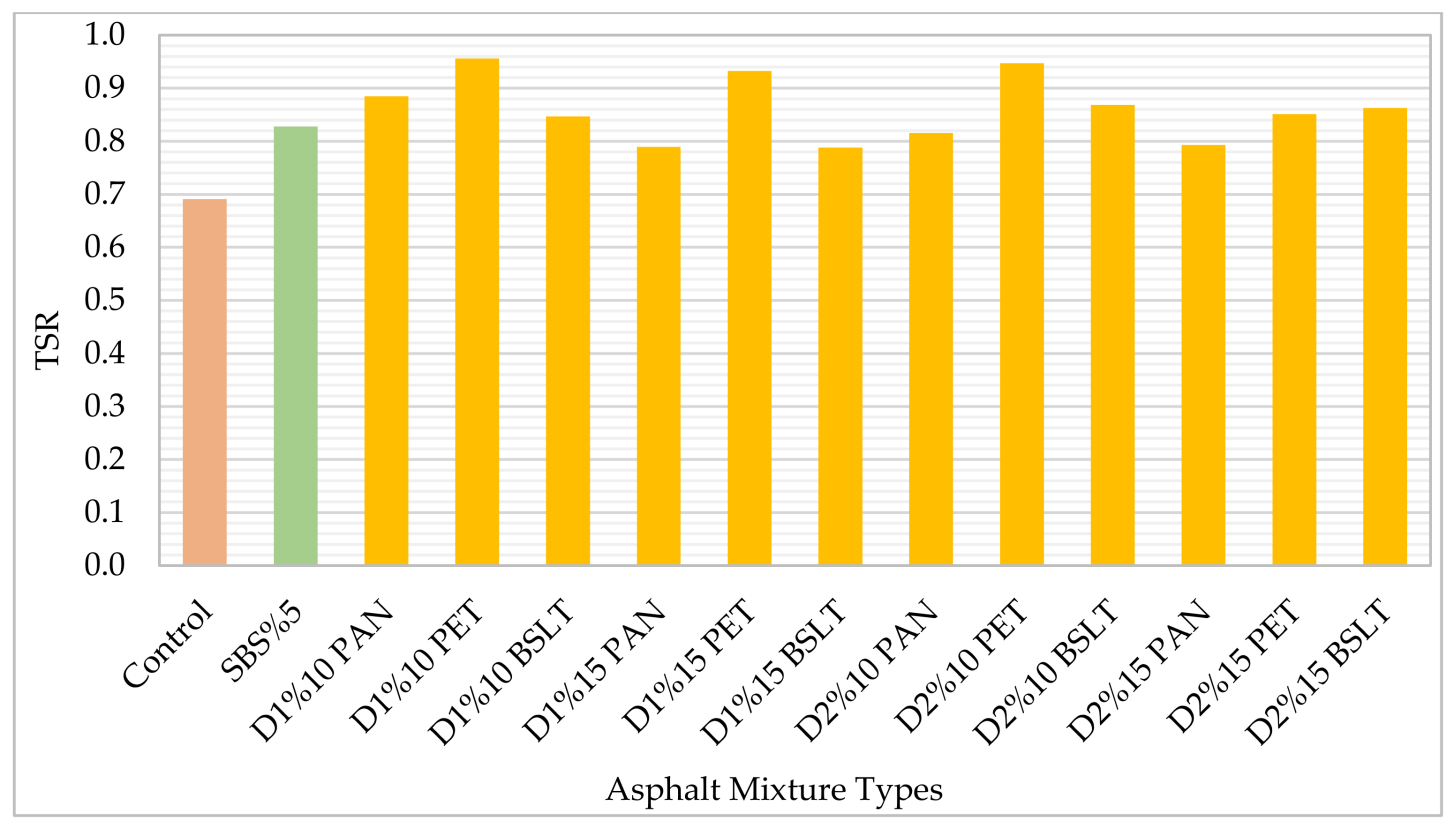
Comparison of the TSR values of the control and modified asphalt mixtures. Different colors indicate the control, SBS-modified, and diatomite–microfiber-modified mixture groups.

The moisture susceptibility of the asphalt mixtures was evaluated based on the ITS values obtained from three conditioned and three unconditioned specimens. The TSR was calculated as the ratio of the average ITS of the conditioned specimens to that of the unconditioned specimens. The corresponding ITS and TSR results are presented in Figure 10 and Figure 11, respectively.

According to the results presented in Figure 10 and Figure 11, the conditioned ITS values were lower than the corresponding unconditioned ITS values for all asphalt mixtures. This reduction indicates that moisture conditioning weakened the binder–aggregate interface, resulting in a loss of tensile strength. For the untreated control mixture, the unconditioned and conditioned ITS values were 1634.05 and 1129.08 kPa, respectively, corresponding to a TSR of 69.9%. As this value is below the commonly accepted minimum requirement of 80%, the untreated mixture exhibited inadequate resistance to moisture-induced damage.

The 5% SBS-modified mixture exhibited unconditioned and conditioned ITS values of 1962.83 and 1624.71 kPa, respectively, resulting in a TSR of 82.8%. This result demonstrates that SBS modification improved both the tensile strength and the retention of mechanical properties after moisture conditioning compared with the untreated control mixture. However, the TSR of the SBS-modified mixture was lower than that of several PET fibre-reinforced diatomite hybrid mixtures. This finding suggests that, although SBS effectively enhances the absolute tensile strength of asphalt mixtures, the combined action of diatomite and fibre reinforcement may provide superior resistance to moisture-induced deterioration by improving strength retention after conditioning [90].

Among the mixtures incorporating D1-type diatomite, those reinforced with PET fibres exhibited the highest resistance to moisture-induced damage. The D1%10 PET mixture achieved the highest moisture resistance of all mixtures, with unconditioned and conditioned ITS values of 1810.28 and 1730.33 kPa, respectively, corresponding to a TSR of 95.6%. Similarly, the D1%15 PET mixture exhibited a high TSR of 93.3%, indicating excellent retention of tensile strength after moisture conditioning. In contrast, the D1%15 PAN and D1%15 BSLT mixtures exhibited TSR values of 78.9% and 78.8%, respectively, both of which were below the commonly accepted minimum requirement of 80%. These findings suggest that, at a diatomite content of 15%, D1-type diatomite may not provide adequate resistance to moisture-induced damage when combined with fibre types other than PET [91]. Although no microstructural characterization was performed in the present study, the observed behaviour may be attributed to the highly porous and absorptive nature of D1-type diatomite. Increased binder absorption at higher diatomite contents may reduce the effective bitumen film thickness and increase mastic stiffness, which could contribute to the lower moisture resistance [88].

The beneficial effect of PET fibre was also evident in mixtures incorporating D2-type diatomite. The D2%10-PET mixture exhibited excellent resistance to moisture-induced damage, with unconditioned and conditioned ITS values of 1763.52 and 1669.98 kPa, respectively, corresponding to a TSR of 94.7%. The D2%15-PET and D2%15-BSLT mixtures also exhibited satisfactory moisture resistance, with TSR values of 85.1% and 86.3%, respectively, both exceeding the commonly accepted minimum requirement of 80%. In contrast, the D2%15-PAN mixture exhibited a TSR of 79.3%, indicating inadequate resistance to moisture-induced damage. These findings suggest that PAN fibre may be less effective than PET fibre in maintaining tensile strength at higher D2-type diatomite contents [92].

When the results were evaluated according to fibre type, PET fibre consistently produced the highest and most stable TSR values across both diatomite types and additive contents. The superior moisture resistance of the PET fibre-reinforced mixtures may be attributed to the combined effects of their relatively hydrophobic nature, favourable interaction with the asphalt binder, enhanced binder retention, and crack-bridging capability. These mechanisms are expected to reduce moisture ingress at the fibre–binder interface, promote a more stable binder film around the aggregates, reinforce the asphalt mastic, and improve stress transfer within the mixture, thereby reducing interfacial deterioration and tensile strength loss under moisture conditioning. Similar observations have been reported in previous studies on PET fibre-modified asphalt mixtures [23,26,27]. In addition, the more stable binder film surrounding the aggregates and fibres is expected to hinder water diffusion through the asphalt mastic, thereby preserving the adhesive bond at the aggregate–binder interface during moisture conditioning [23,26,27]. Conversely, excessive diatomite contents may increase binder absorption and reduce the effective binder film thickness, facilitating moisture ingress and contributing to the lower TSR values observed for several mixtures containing 15% diatomite [88].

The results of the moisture susceptibility tests demonstrate that the diatomite–fibre hybrid modification numerically increased the moisture resistance of the asphalt mixtures compared with the untreated control mixture. However, the extent of this improvement depended on the diatomite type, fibre type, and additive content. The D1%10-PET and D2%10-PET mixtures exhibited the highest TSR values, indicating that a diatomite content of 10% combined with PET fibre provided the most effective resistance to moisture-induced damage. Although increasing the diatomite content to 15% maintained satisfactory moisture resistance in PET fibre-reinforced mixtures, it reduced the moisture resistance of mixtures reinforced with PAN fibre and, to a lesser extent, basalt fibre. These findings indicate that optimum moisture resistance can be achieved not only through the incorporation of diatomite and fibres but also through the appropriate combination of diatomite type, additive content, and fibre type.

It should be noted that a high unconditioned ITS does not necessarily correspond to a high TSR. While the unconditioned ITS reflects the initial tensile strength of the asphalt mixture, the TSR represents the ability of the mixture to retain this strength after moisture conditioning, as determined in accordance with AASHTO T283-21 [65]. Therefore, mixtures with relatively high initial ITS values may still exhibit lower TSR values if they experience a greater reduction in tensile strength due to moisture-induced deterioration at the aggregate–binder interface [67,68].

### 3.3. Cantabro Loss Test Results and Discussion

**Figure 12:**
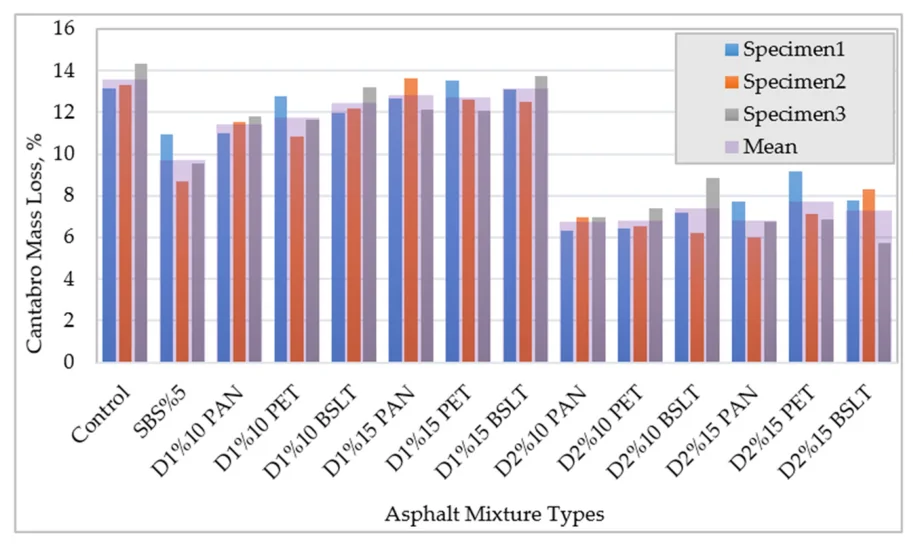
Individual Cantabro loss values for the replicate specimens of each asphalt mixture.

**Figure 13:**
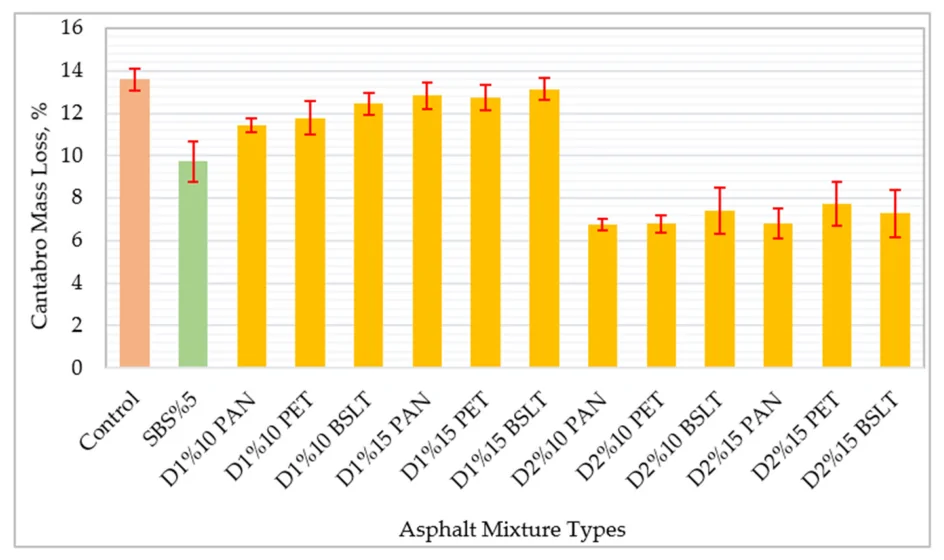
Average Cantabro loss values of the asphalt mixtures. Different colors indicate the control, SBS-modified, and diatomite–microfiber-modified mixture groups.

The Cantabro loss test was performed to evaluate the resistance of the asphalt mixtures to raveling under impact and abrasion. Lower Cantabro loss values indicate better cohesion within the asphalt mixture, reflecting improved aggregate–binder adhesion and greater resistance to particle disintegration. Three replicate specimens were tested for each mixture, and the corresponding Cantabro loss results are presented in Figure 12 and Figure 13.

According to the experimental results presented in Figure 12 and Figure 13, the untreated control mixture exhibited the highest Cantabro loss among all the asphalt mixtures. The average Cantabro loss of the control mixture was 13.58%, indicating relatively poor resistance to ravelling under impact and abrasion. This behaviour suggests that, in the absence of modifiers, the binder–aggregate system provided limited cohesion and was less effective in maintaining aggregate integrity during mechanical loading. In contrast, the average Cantabro loss of the 5% SBS-modified mixture decreased to 9.73%, demonstrating that SBS modification enhanced the cohesion and elasticity of the asphalt binder, thereby reducing the detachment of aggregate particles. Nevertheless, the SBS-modified mixture exhibited higher Cantabro loss than several hybrid mixtures incorporating D2-type diatomite and fibre reinforcement, indicating that the combined modification strategy provided superior resistance to ravelling.

Among the hybrid-modified mixtures, the Cantabro loss of the D1%10 mixtures was 11.44%, 11.77%, and 12.45% for the PAN, PET, and basalt fibre-reinforced mixtures, respectively. Although these values were lower than that of the untreated control mixture, they remained higher than that of the 5% SBS-modified mixture. In contrast, the D2%15 mixtures exhibited substantially lower Cantabro loss values of 6.82%, 7.73%, and 7.28% for the PAN, PET, and basalt fibre-reinforced mixtures, respectively. These results indicate that D2-type diatomite provided greater resistance to ravelling than D1-type diatomite when incorporated into the hybrid modification system. When the results were evaluated according to fibre type, the mixtures incorporating D2-type diatomite consistently exhibited lower Cantabro loss values than their D1 counterparts, regardless of the fibre type. The lowest Cantabro loss was observed for the D2%10-PAN mixture, followed by the D2%10-PET and D2%15-PAN mixtures. These findings suggest that the combination of D2-type diatomite and fibre reinforcement effectively improved the cohesion of the asphalt mixture and enhanced its resistance to ravelling. The beneficial effect of fibre reinforcement may be associated with the formation of a three-dimensional reinforcing network that bridges microcracks, facilitates stress transfer, and helps maintain the integrity of the aggregate–binder structure. Previous studies have likewise reported that fibre reinforcement improves the mechanical strength, fatigue resistance, crack resistance, and stress distribution of asphalt mixtures [23].

It has also been reported that diatomite can reinforce the asphalt mastic and enhance the mechanical performance of asphalt mixtures. Yang et al. [93] reported that the high specific surface area and porous structure of diatomite modify the rheological behaviour of the asphalt binder, thereby improving mixture performance. Similarly, Cheng et al. [31] demonstrated that hybrid modification incorporating diatomite and basalt fibre can significantly enhance the mechanical performance and durability of asphalt mixtures.

The Cantabro loss results demonstrate that hybrid modification had a pronounced effect on the ravelling resistance of the asphalt mixtures. The untreated control mixture exhibited the highest Cantabro loss, whereas SBS modification reduced material loss by improving the cohesion of the asphalt binder. However, the lowest Cantabro loss values were achieved by the hybrid mixtures incorporating D2-type diatomite and fibre reinforcement. Although the D1%10 mixtures exhibited lower Cantabro loss than the untreated control mixture, their performance remained inferior to that of the 5% SBS-modified mixture. In contrast, the D2%15 mixtures exhibited the lowest Cantabro loss values, ranging from 6.82% to 7.73%, indicating excellent resistance to ravelling under impact and abrasion. These findings suggest that the combined use of D2-type diatomite and fibre reinforcement provides the most effective hybrid modification strategy for improving the durability of asphalt mixtures against particle loss.

### 3.4. Repeated Load Creep Test Results and Discussion

**Figure 14:**
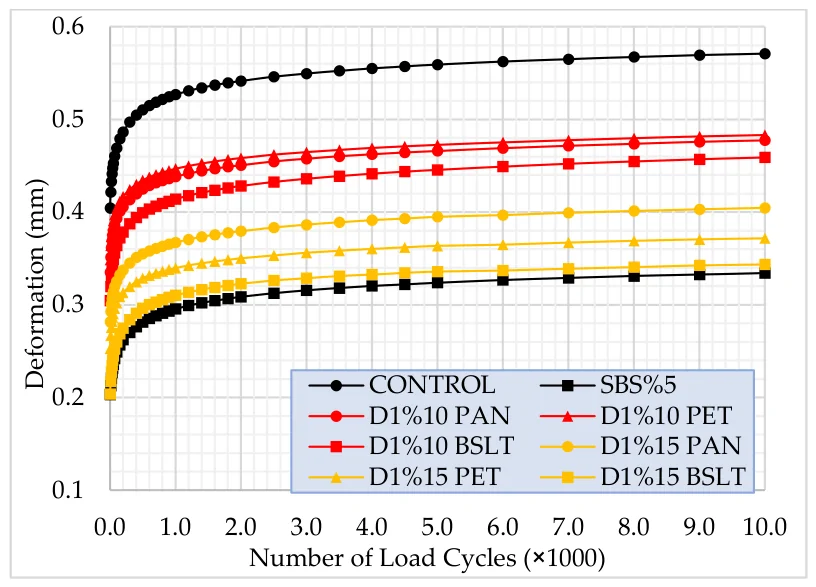
Repeated load creep curves of asphalt mixtures modified with D1-type diatomite and different fibre types.

**Figure 15:**
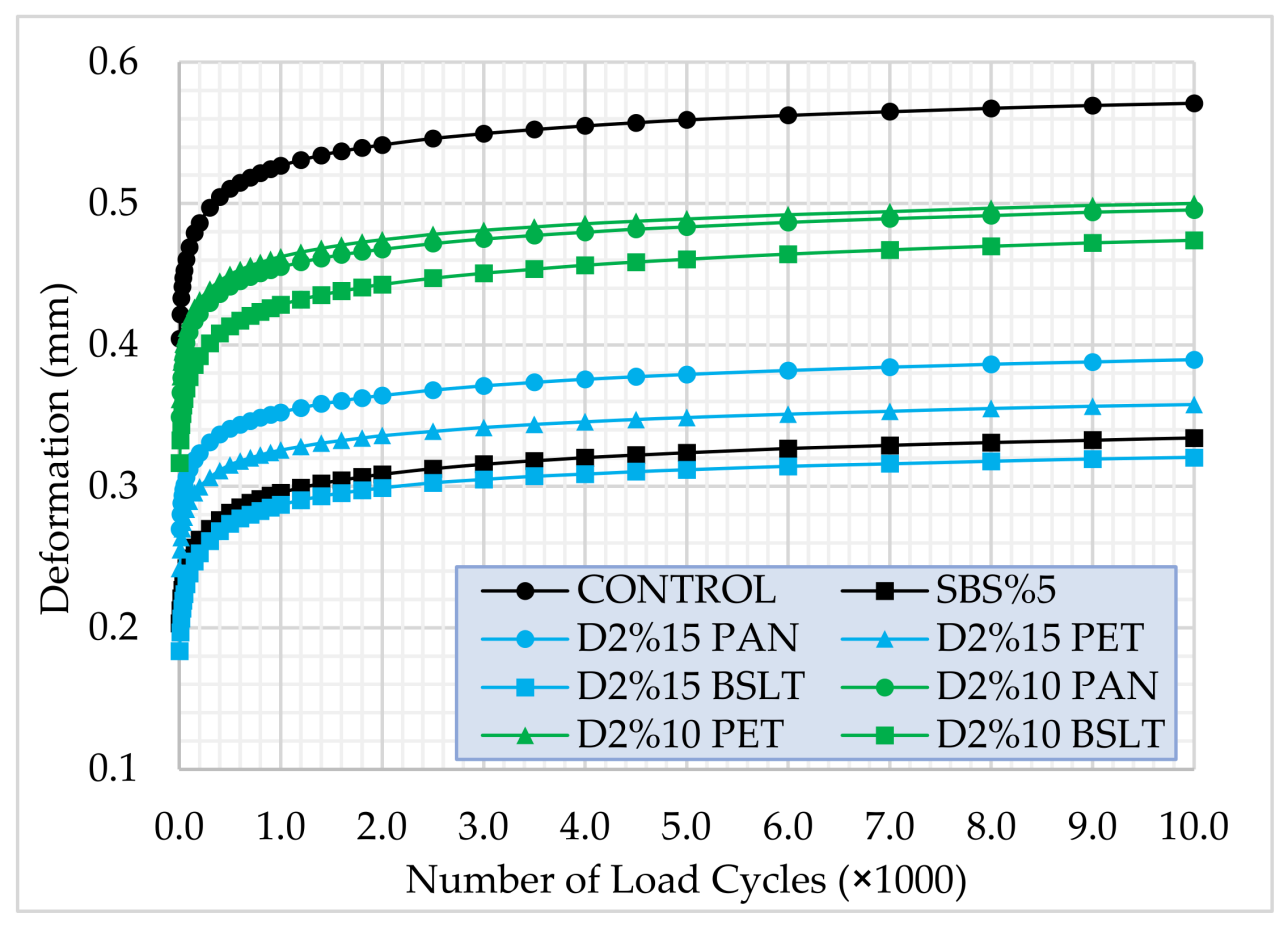
Repeated load creep curves of asphalt mixtures modified with D2-type diatomite and different fibre types.

Figure 14 and Figure 15 present the deformation–load cycle curves obtained from the repeated load creep tests performed on the control and modified asphalt mixtures. For each mixture, the test was conducted on three replicate specimens, and the curves shown in the figures represent the arithmetic mean of the deformation responses obtained from the three specimens.

Overall, the test results showed that all asphalt mixtures exhibited a rapid increase in deformation during the initial stage of repeated loading, followed by a gradual reduction in the deformation rate until the creep curves approached a nearly stable plateau. This behaviour is consistent with the viscoelastic response of asphalt mixtures. Under repeated loading, the rapid deformation at the initial stage may be associated with the rearrangement of the aggregate skeleton, the compaction of the asphalt mastic, and the viscous deformation of the asphalt binder. As the number of load cycles increased, the internal structure of the mixtures became progressively more stable, resulting in a lower rate of permanent deformation accumulation. Such permanent deformation under elevated-temperature loading conditions is commonly associated with rutting and is governed by the shear resistance of the mixture, the viscous flow characteristics of the asphalt binder, and the interlocking capacity of the aggregate skeleton [6].

The untreated control mixture exhibited the highest deformation among all mixtures. After 10,000 load cycles, the control mixture reached deformation of approximately 0.57 mm, indicating a greater susceptibility to permanent deformation under repeated loading than the modified mixtures. In contrast, all modified mixtures exhibited lower deformation than the untreated control mixture. These results indicate that the incorporation of SBS, diatomite, and fibre reinforcement effectively restricted the viscous flow of the asphalt binder and enhanced the resistance of the mixtures to permanent deformation under repeated loading.

The 5% SBS-modified mixture exhibited one of the lowest deformation values among all the mixtures. This behaviour may be associated with the elastomeric network formed by SBS within the asphalt binder, which enhances the elastic recovery of the binder after loading and improves its resistance to permanent deformation under elevated-temperature conditions.

Among the hybrid-modified mixtures, those containing 15% diatomite generally exhibited lower deformation than the corresponding mixtures containing 10% diatomite. This improvement may be attributed to the highly porous structure, large specific surface area, and high bitumen absorption capacity of diatomite. By absorbing a portion of the asphalt binder, diatomite increases the viscosity and stiffness of the asphalt mastic, thereby restricting viscous flow and enhancing the resistance of the mixtures to permanent deformation under repeated loading. Previous studies have likewise reported that diatomite modification improves the high-temperature stability and rutting resistance of asphalt mixtures [31].

Among the hybrid-modified mixtures, the D2%15 BSLT, D1%15 BSLT, D2%15 PET, and D1%15 PET mixtures exhibited the lowest deformation under repeated loading. The superior performance of these mixtures may be associated with the combined effects of the stiffening action of diatomite and the reinforcing effect of the fibres. The fibres also contribute to the confinement of the aggregate skeleton, limiting particle movement under repeated loading and promoting more effective aggregate interlocking. Consequently, localized stresses are redistributed more uniformly throughout the mixture, reducing the accumulation of permanent deformation [23,30,31,32]. In particular, the basalt fibre-reinforced mixtures exhibited remarkably low deformation. Owing to their high elastic modulus, excellent thermal stability, and mineral-based structure, basalt fibres can effectively improve load transfer within the asphalt mixture and enhance the stability of the aggregate skeleton. Cheng et al. [31] likewise reported that the combined use of diatomite and basalt fibre improves the high-temperature stability, moisture resistance, and overall pavement performance of asphalt mixtures. Similarly, Davar et al. [30] and Zhu et al. [32] demonstrated that the combined incorporation of basalt fibre and diatomite enhances the fatigue performance of hot mix asphalt.

The performance of the PAN- and PET fibre-reinforced mixtures was influenced by the diatomite content. The mixtures containing 10% diatomite generally exhibited higher deformation than their corresponding 15% diatomite counterparts, whereas increasing the diatomite content to 15% resulted in a noticeable reduction in permanent deformation. These findings indicate that the effectiveness of fibre reinforcement depends not only on the fibre type but also on the type and content of diatomite. Fibre reinforcement forms a three-dimensional reinforcing network within the asphalt mixture, facilitating stress transfer and delaying crack initiation and propagation. However, its contribution to permanent deformation resistance is influenced by fibre characteristics, fibre dispersion, and the quality of the fibre–binder interface [23]. The lower deformation observed in the mixtures containing 15% diatomite further suggests that the stiffening effect of diatomite on the asphalt mastic plays a dominant role in improving resistance to permanent deformation under repeated loading.

Overall, the repeated load creep results demonstrate that hybrid diatomite–fibre modification improves the high-temperature performance of asphalt mixtures through the complementary effects of the two modifiers. Diatomite increases the viscosity and stiffness of the asphalt mastic, thereby enhancing resistance to permanent deformation, whereas the fibres reinforce the mixture by improving load transfer and maintaining structural integrity. The combined action of these mechanisms contributes to reduced permanent deformation under repeated loading and improved rutting resistance compared with the unmodified control mixture.

The repeated load creep results showed that the untreated control mixture exhibited the greatest permanent deformation, whereas the 5 wt.% SBS-modified mixture and several hybrid mixtures containing 15% diatomite exhibited comparatively lower accumulated deformation. In general, increasing the diatomite content from 10% to 15% reduced permanent deformation, consistent with the ANOVA results indicating that diatomite content was the primary factor influencing creep performance. Several hybrid mixtures achieved permanent deformation resistance numerically comparable to that of the SBS-modified mixture.

To provide a more quantitative evaluation of the repeated load creep behaviour, the creep slopes were calculated for each asphalt mixture. The creep slope was determined using the deformation–load cycle curves over the range of 3000–10,000 load cycles, excluding the initial stage characterised by rapid deformation accumulation. This interval corresponds to the approximately linear secondary creep region and was therefore considered suitable for comparing the rate of permanent deformation accumulation under repeated loading.

The creep slope was calculated using Equation (7). The calculated creep slope values for all asphalt mixtures are presented in Table 6.

(7) $$Creep\;slope=\frac{d_{10,000}-d_{3000}}{10,000-3000}$$

where d_10,000_ and d_3000_ denote the accumulated deformation measured at 10,000 and 3000 load cycles, respectively.

Table 6 shows that the lowest creep slope within the 3000–10,000 load cycle interval was obtained for the D1%15 BSLT mixture, followed by the D2%15 BSLT, D1%15 PET, and D2%15 PET mixtures. These results indicate that the basalt fibre- and PET fibre-reinforced mixtures containing 15% diatomite were more effective in limiting the rate of permanent deformation accumulation during the secondary creep stage.

In contrast, the highest creep slope values were obtained for the D2%10 BSLT, D1%10 BSLT, and untreated control mixtures. The relatively high creep slope of the control mixture indicates that it exhibited not only greater permanent deformation but also a higher rate of deformation accumulation under repeated loading. Moreover, the comparatively high creep slopes observed for the basalt fibre-reinforced mixtures containing 10% diatomite suggest that the interaction between fibre reinforcement and diatomite was insufficient at the lower diatomite content to effectively suppress permanent deformation accumulation.

Mixtures containing 15% diatomite generally exhibited lower creep slope values than those containing 10% diatomite. This behaviour may be associated with the increased stiffness of the asphalt mastic and the corresponding reduction in viscous flow at the higher diatomite content. These observations are consistent with the ANOVA results, which identified diatomite content as the primary factor influencing permanent deformation resistance.

To further evaluate the repeatability of the repeated load creep test, the three-replicate deformation–load cycle curves for each mixture were analysed together. The area between the two most divergent creep curves was calculated using the trapezoidal rule and was used as an indicator of the overall variability among replicate specimens throughout the test. In addition, the standard deviation of the accumulated deformation at 10,000 load cycles was calculated for each mixture. Lower values of both parameters indicate better repeatability and greater consistency of the experimental results.

Among all mixtures, the D1%10 PAN mixture exhibited the smallest area between the creep curves as well as the lowest standard deviation, indicating the most consistent deformation behaviour under repeated loading. In contrast, the D2%15 PAN mixture exhibited the largest area between the creep curves and the highest standard deviation, suggesting greater variability in the deformation responses of the replicate specimens.

### 3.5. Evaluation of the SCB Test Results

**Figure 16:**
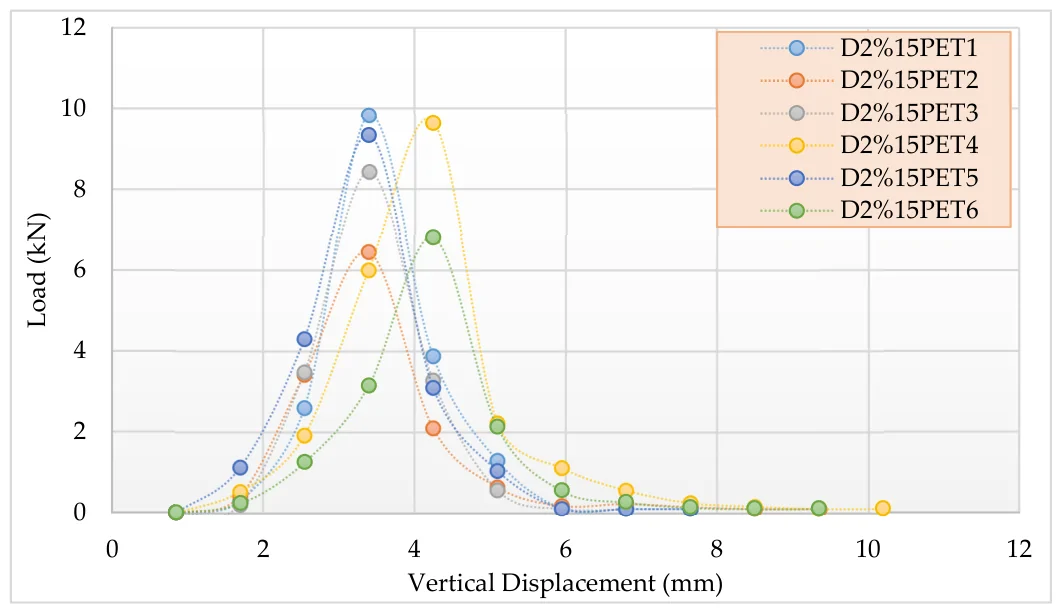
Representative load–vertical displacement curves obtained from the SCB test.

**Figure 17:**
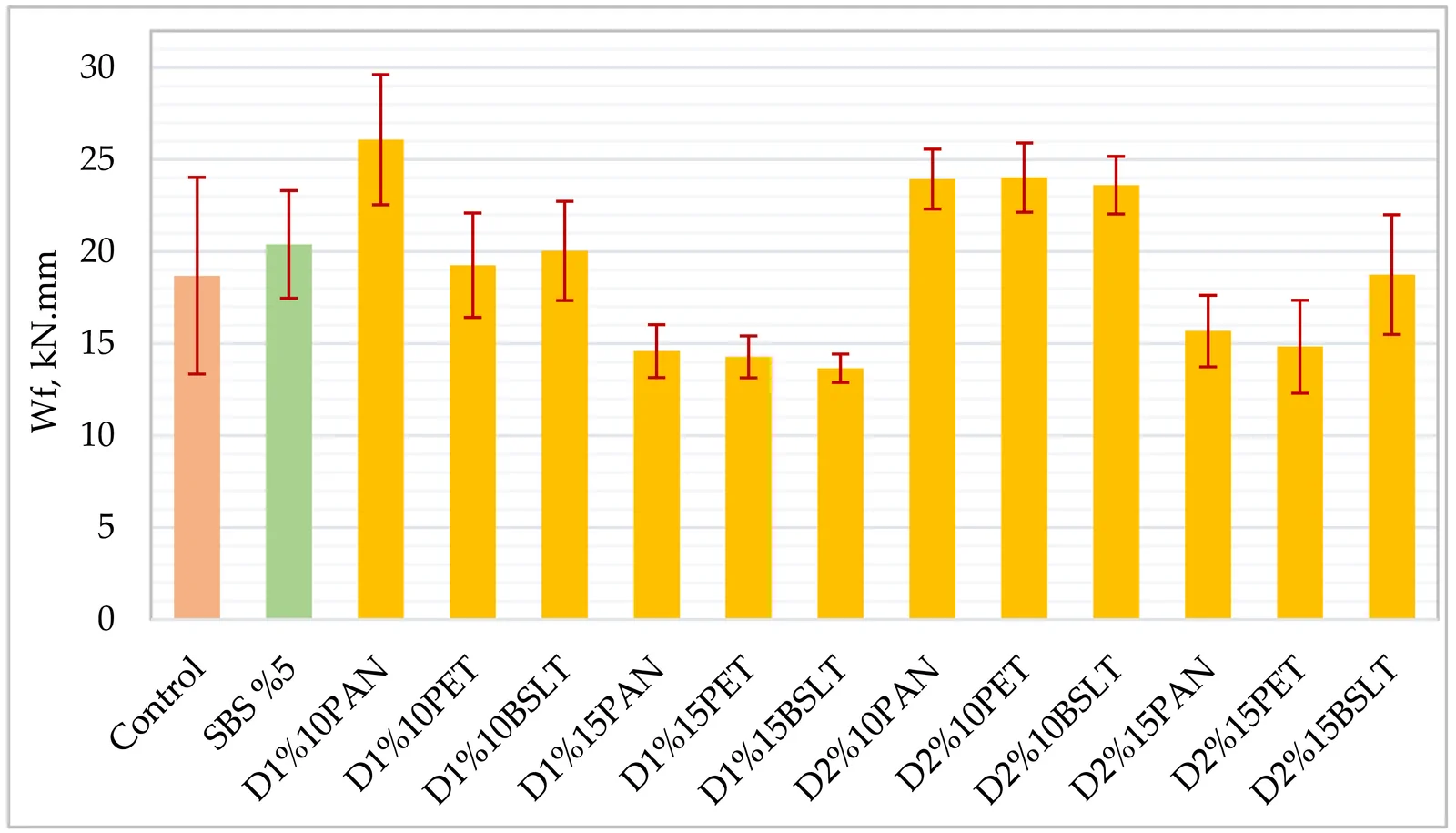
Comparison of the W_f_ values of the control and modified asphalt mixtures. Different colors indicate the control, SBS-modified, and diatomite–microfiber-modified mixture groups.

**Figure 18:**
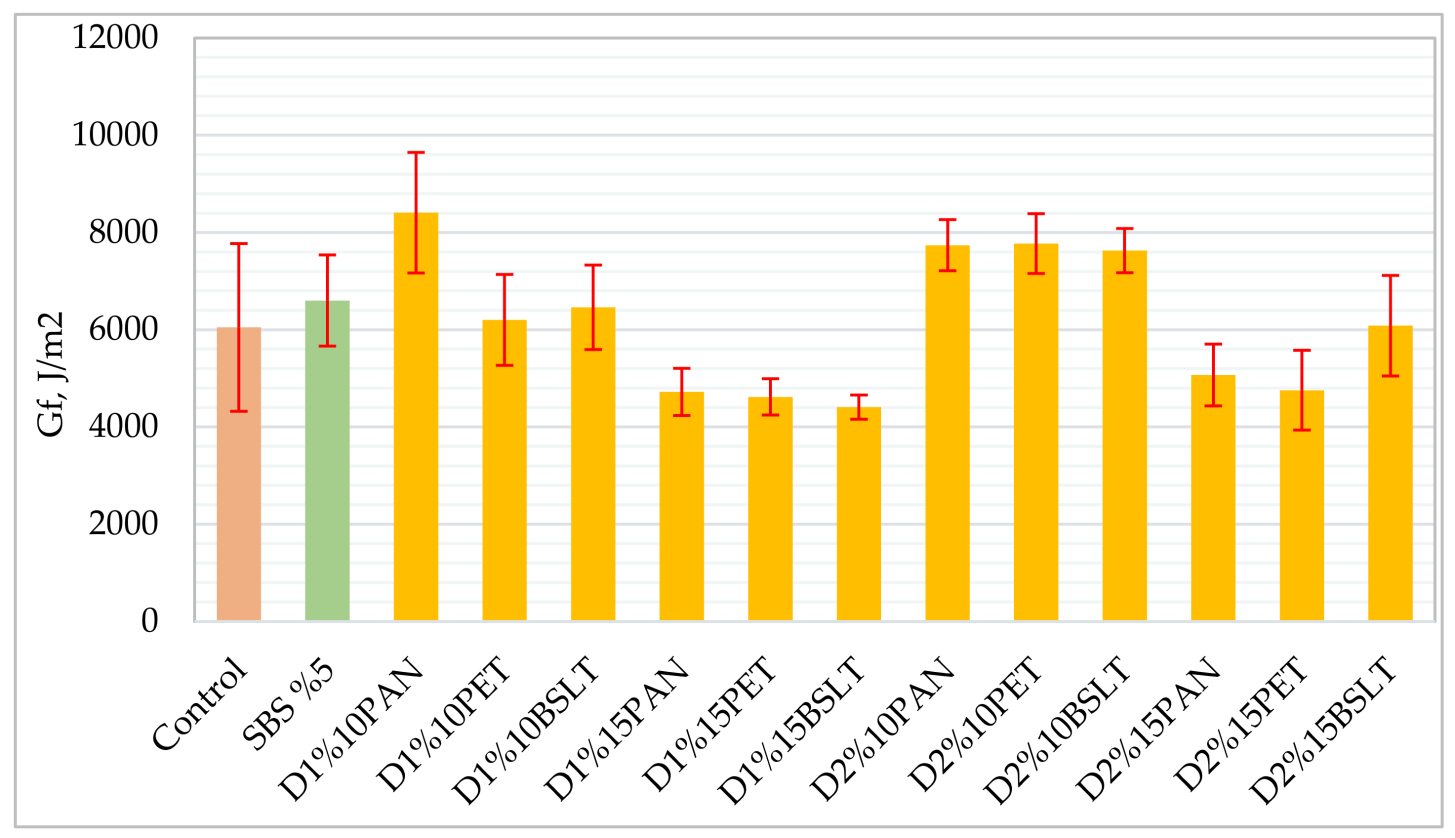
Comparison of the G_f_ values of the control and modified asphalt mixtures. Different colors indicate the control, SBS-modified, and diatomite–microfiber-modified mixture groups.

**Figure 19:**
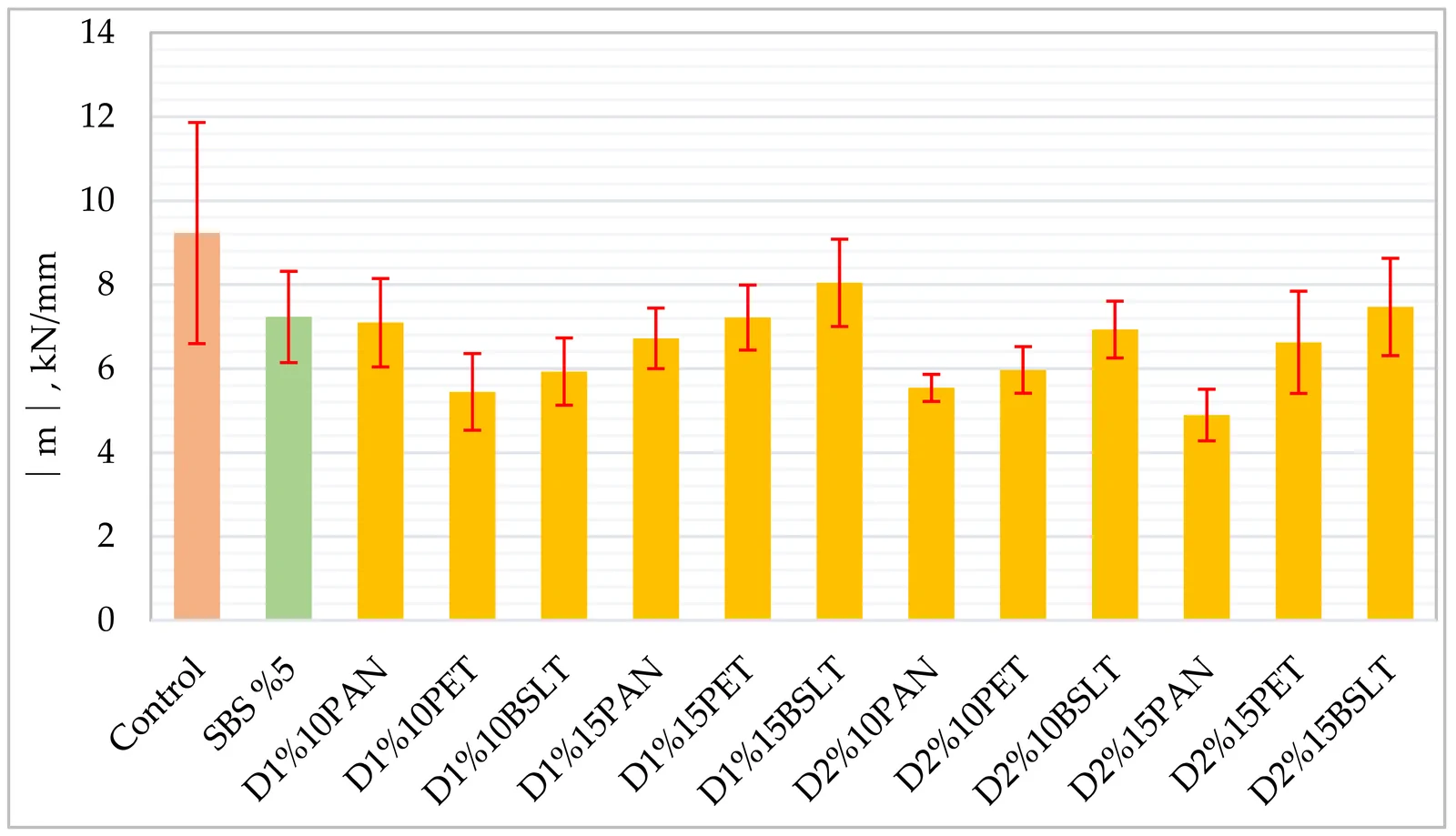
Comparison of the |m| values of the control and modified asphalt mixtures. Different colors indicate the control, SBS-modified, and diatomite–microfiber-modified mixture groups.

**Figure 20:**
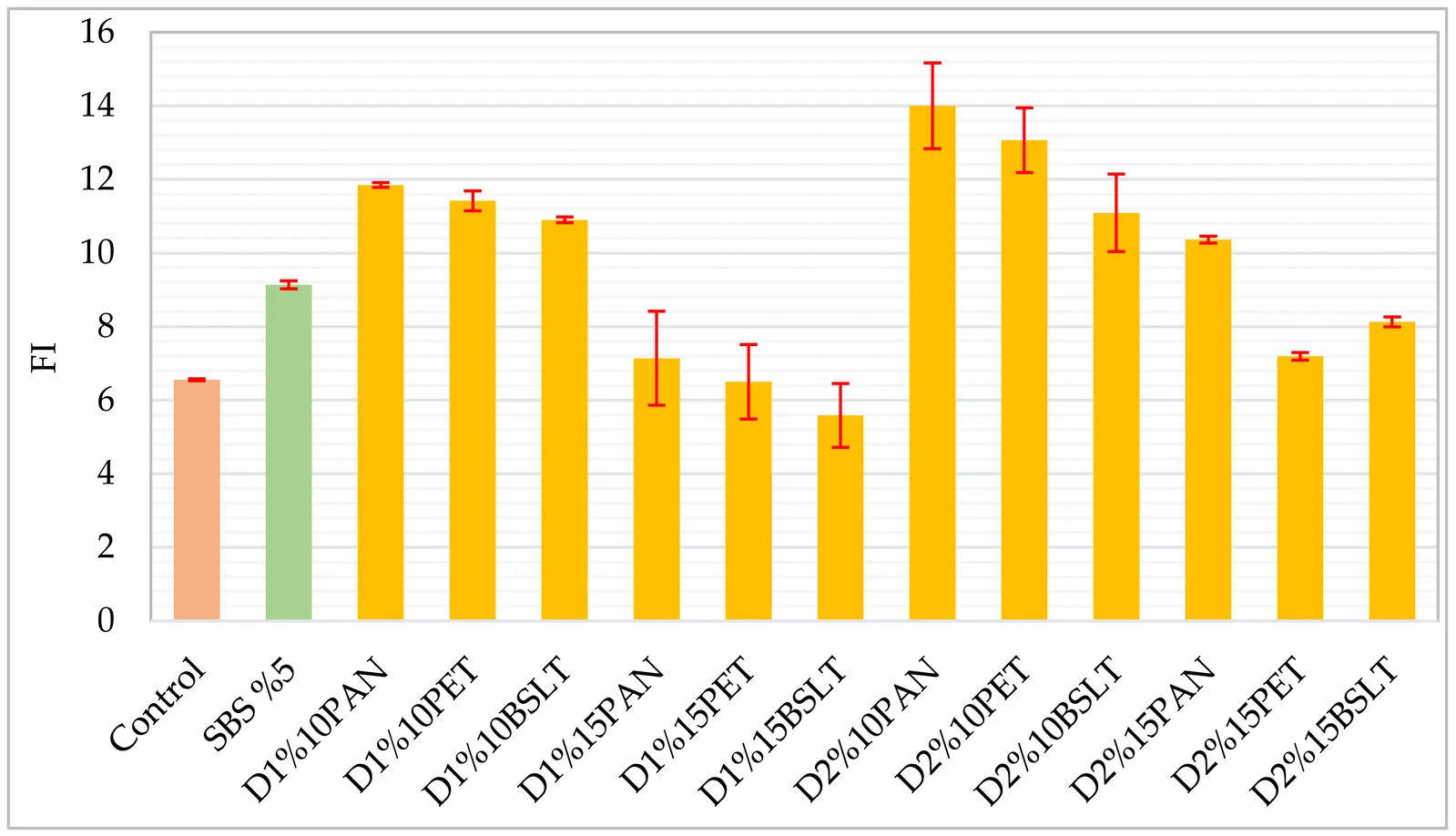
Comparison of the FI values of the control and modified asphalt mixtures. Different colors indicate the control, SBS-modified, and diatomite–microfiber-modified mixture groups.

SCB test was performed on the control and modified asphalt mixture specimens using six replicate specimens for each mixture. The load–displacement curves obtained from the tests were used to calculate the W_f_, G_f_, |m|, and FI. Representative load–displacement curves obtained from the SCB tests are presented in Figure 16, whereas the variations in W_f_, G_f_, |m|, and FI for the different asphalt mixtures are shown in Figure 17, Figure 18, Figure 19 and Figure 20, respectively.

The SCB test parameters provide complementary information on the crack initiation and crack propagation resistance of asphalt mixtures. According to Ozer et al. [79], the G_f_ represents the energy required for crack initiation, whereas the FI, together with the post-peak |m|, characterizes crack propagation resistance and fracture ductility.

The untreated control mixture exhibited the lowest crack propagation resistance, with an FI value of approximately 6.5, whereas SBS modification increased the FI value to approximately 9.0. This improvement is consistent with previous studies reporting that polymer modification enhances the cracking resistance of asphalt mixtures [94]. In addition, the |m| value decreased from approximately 9 kN/mm for the control mixture to approximately 7 kN/mm after SBS modification, indicating a more gradual reduction in load-carrying capacity after the peak load and, consequently, a more ductile fracture behaviour. Rivera-Perez et al. [95] likewise reported that lower post-peak slope values are associated with higher FI values and improved crack propagation resistance.

For the D1 series, mixtures containing 10% diatomite exhibited FI values ranging from 10.9 to 11.8, representing a substantial improvement compared with the untreated control mixture. Among these mixtures, D1%10 PAN exhibited the highest fracture energy, reaching approximately 8300 J/m^2^. Increasing the diatomite content to 15% resulted in a marked reduction in both FI and Gf, with FI values decreasing to 5.5–7.0 and fracture energy decreasing to approximately 4400–4700 J/m^2^. The reduction in fracture energy indicates that less energy was required to initiate cracking, reflecting a transition toward a more brittle fracture response. Ozer et al. [79] similarly reported that higher FI and G_f_ values correspond to greater resistance to crack propagation.

A similar trend was observed for the D2 series. All mixtures containing 10% diatomite exhibited relatively high G_f_ and FI values. Among all mixtures evaluated, D2%10 PAN achieved the highest flexibility index, with an FI value of approximately 14. This mixture also exhibited a fracture energy of approximately 7700 J/m^2^ and a relatively low |m| value of approximately 5.5 kN/mm. The low post-peak slope indicates that the load-carrying capacity decreased more gradually after peak loading, resulting in slower crack propagation. According to the flexibility index approach proposed by Ozer et al. [79], the combination of high fracture energy and a low post-peak slope corresponds to higher FI values and reflects a more ductile fracture behaviour. Consequently, the D2%10 PAN mixture exhibited the greatest resistance to crack propagation among all mixtures investigated.

Increasing the diatomite content to 15% also reduced the FI values of the D2 series. The FI values of the D2%15 PAN, D2%15 PET, and D2%15 BSLT mixtures were approximately 10.3, 7.1, and 8.0, respectively, accompanied by corresponding reductions in fracture energy. Nevertheless, the FI values of most of these mixtures remained higher than that of the untreated control mixture. These findings suggest that excessive diatomite content may adversely affect cracking resistance and that the optimum additive content should not exceed a certain level.

Overall, the SCB test results demonstrate that the combination of high G_f_, high FI, and low |m| provides superior resistance to crack propagation. Among the mixtures investigated, D2%10 PAN exhibited the best overall fracture performance, followed by D2%10 PET, D2%10 BSLT, and D1%10 PAN. In contrast, increasing the diatomite content from 10% to 15% generally reduced both the fracture energy and flexibility index of the mixtures. These findings are consistent with previous studies reporting that high fracture energy, high flexibility index, and low post-peak slope values are indicative of improved cracking resistance in asphalt mixtures [79,95].

In addition to the crack-bridging effect, fibre reinforcement is expected to delay crack initiation by reducing local stress concentrations and to impede crack propagation through crack arrest mechanisms. The improved fracture performance may also be associated with greater energy dissipation resulting from fibre pull-out and fibre–binder debonding during crack propagation. Although these mechanisms were not directly examined in the present study, they are consistent with the higher fracture energy and flexibility index values obtained for the fibre-reinforced mixtures and have been widely reported in previous studies [23,79,95].

The improved performance of the hybrid-modified mixtures cannot be attributed solely to the stiffening effect of diatomite. While diatomite increased the stiffness and stability of the asphalt mastic, the fibre treatments likely improved load transfer, restricted crack propagation through crack-bridging mechanisms, and redistributed localized stresses. The experimental results therefore suggest that the combined use of diatomite and fibres provided complementary reinforcing mechanisms rather than a simple stiffness increase alone. This complementary behaviour is consistent with the superior tensile and fracture performance observed for the PAN- and PET-treated mixtures relative to the SBS-modified reference mixture.

### 3.6. Statistical Evaluation

The effects of diatomite type, diatomite content, and fibre type on the performance of the asphalt mixtures were evaluated using three-way ANOVA, and the corresponding results are summarized in Table 7. The statistical analysis indicated that the relative importance of the investigated factors varied depending on the performance parameter considered.

For ITS, significant main effects were observed for both diatomite type (*p* < 0.001) and diatomite content (*p* = 0.018), whereas the effect of fibre type did not reach the 5% significance level (*p* = 0.053). None of the interaction terms were statistically significant, indicating that the effects of diatomite type and content on ITS were generally consistent across the different fibre types.

For permanent deformation measured after 10,000 load cycles, only diatomite content had a statistically significant effect (*p* < 0.001). Neither diatomite type, fibre type, nor any interaction term was significant, suggesting that differences in permanent deformation were governed primarily by the amount of diatomite incorporated into the mix.

For Cantabro loss, only diatomite type exhibited a statistically significant main effect (*p* < 0.001), whereas diatomite content, fibre type, and all interaction terms were statistically insignificant. These results indicate that the difference between the D1 and D2 diatomites was the principal factor influencing the ravelling resistance of the asphalt mixtures.

For the G_f_ obtained from the SCB test, both diatomite type and diatomite content had significant main effects. In addition, the significant diatomite type × fibre type, diatomite content × fibre type, and three-way interaction effects indicate that the influence of fibre reinforcement on fracture energy depended on both the type and content of diatomite.

For the |m|, all three main factors—diatomite type, diatomite content, and fibre type—were statistically significant. Furthermore, the interactions between diatomite type and content, diatomite type and fibre type, and diatomite content and fibre type were also significant. Similarly, for the FI, all main factors were significant, together with the diatomite type × fibre type, diatomite content × fibre type, and three-way interaction effects. These findings indicate that the effects of fibre type on cracking performance varied according to the diatomite type and content. Therefore, fracture performance should be interpreted based on the specific combination of the investigated factors rather than on the main effects alone. Instead, the fracture behaviour of the asphalt mixtures depended on the combined effects of diatomite type, diatomite content, and fibre type, highlighting the importance of evaluating these variables as an integrated hybrid modification system.

The effects of mixture type and conditioning state on ITS were evaluated using a two-way ANOVA. Both mixture type and conditioning state had statistically significant effects on ITS (*p* < 0.001). In contrast, the interaction between mixture type and conditioning state was not statistically significant (*p* = 0.787). These results indicate that although the mixtures differed significantly in their overall tensile strength levels, the reduction in ITS caused by moisture conditioning was statistically similar across all mixture types. Consequently, the TSR was considered a descriptive indicator of moisture resistance, providing a comparative measure of the moisture susceptibility of the investigated asphalt mixtures rather than evidence of statistically different moisture-conditioning responses among the mixtures.

## 4. Conclusions

This study comprehensively evaluated the performance of hybrid-modified asphalt mixtures incorporating two diatomite types (D1 and D2), two diatomite contents (10% and 15%), and three fibre types (PAN, PET, and basalt). Based on the experimental results and statistical analyses, the following conclusions can be drawn:

Factorial ANOVA demonstrated that the dominant factors governing mixture performance varied according to the evaluated property. ITS was significantly affected by diatomite type and diatomite content, whereas permanent deformation was primarily influenced by diatomite content. Cantabro loss was significantly affected by diatomite type, while significant interaction effects for the SCB parameters indicated that fracture performance depended on the combined effects of diatomite type, diatomite content, and fibre type. Moisture conditioning significantly reduced ITS; however, the reduction was statistically similar for all mixtures. Among all investigated mixtures, the D1%10 PAN mixture exhibited the highest indirect tensile strength (4712.36 kPa), corresponding to improvements of 43.8% and 22.4% compared with the control and 5 wt.% SBS-modified mixtures, respectively. These findings indicate that the combined use of D1-type diatomite and PAN fibres provided the most effective reinforcement against low-temperature tensile loading and demonstrated that the proposed hybrid modification system can provide greater tensile strengthening than conventional SBS modification when an appropriate combination of diatomite type and fibre treatment is selected.

Among the tested mixtures, PET fibre generally exhibited the highest TSR values. The D1%10 PET mixture achieved the highest moisture resistance, with a TSR of 0.956. Increasing the diatomite content to 15% reduced the TSR below the commonly accepted 80% threshold for several mixtures, suggesting that excessive diatomite addition may adversely affect moisture resistance. Among the tested mixtures, PET fibre generally exhibited the highest TSR values. The D1%10 PET mixture achieved the highest moisture resistance, with a TSR of 0.956, representing an approximately 37% increase in TSR compared with the untreated control mixture (TSR = 0.699) and exceeding the TSR of the 5 wt.% SBS-modified reference mixture (TSR = 0.828). Increasing the diatomite content to 15% reduced the TSR below the commonly accepted 80% threshold for several mixtures, suggesting that excessive diatomite addition may adversely affect moisture resistance.

Diatomite content was the primary factor governing permanent deformation resistance. Mixtures containing 15% diatomite consistently exhibited lower accumulated permanent deformation and lower secondary creep slopes than those containing 10% diatomite, confirming the beneficial effect of increased diatomite content on rutting resistance. Although the D1%15 BSLT and D2%15 BSLT mixtures exhibited the lowest creep slopes among all investigated mixtures, the statistical analysis indicated that fibre type and diatomite type had no significant effect on permanent deformation.

The SCB results demonstrated that fracture performance was influenced by the interaction between diatomite type, diatomite content, and fibre type. The D2%10 PAN mixture exhibited the highest flexibility index (FI = 14.003), representing more than a twofold increase compared with the untreated control mixture (FI = 6.6), whereas the D1%10 PAN mixture achieved the highest fracture energy (G_f_ = 8405 J/m^2^). These results indicate that the combined use of 10% diatomite and fibre reinforcement substantially enhanced crack propagation resistance. However, because significant interaction effects were observed, these findings should be interpreted for the specific hybrid combinations evaluated rather than as evidence of the overall superiority of any individual fibre type.

The hybrid diatomite–fibre mixtures exhibited performance numerically comparable to, and in some cases greater than, that of the 5 wt.% SBS-modified reference mixture for certain performance indicators. In particular, the D2%15 PAN mixture reduced Cantabro loss from 13.58% for the untreated control mixture to 6.82%, corresponding to an approximately 50% reduction in particle loss. Several D2-based hybrid mixtures also exhibited lower Cantabro loss and higher flexibility index values than the SBS-modified reference mixture. These comparisons are descriptive and do not imply statistically significant differences between the hybrid mixtures and the SBS-modified reference.

Although a detailed cost–performance analysis was beyond the scope of the present study, the investigated hybrid modification system may represent a practical alternative to conventional SBS modification. Diatomite is an abundant and relatively low-cost natural mineral, while recycled PET fibres provide an environmentally sustainable reinforcement option through the utilization of waste plastic materials. Nevertheless, comprehensive cost–performance and life-cycle cost analyses are required before large-scale practical implementation of these hybrid-modified asphalt mixtures.

The effectiveness of fibre reinforcement depended on the diatomite content. PAN fibres provided the greatest improvements in tensile strength and fracture performance when combined with 10% diatomite, whereas basalt fibres were more effective in improving permanent deformation resistance at 15% diatomite. PET fibres consistently exhibited the highest moisture resistance across both diatomite contents, indicating that the optimum hybrid modification strategy depends on the combined selection of fibre type and diatomite content according to the targeted pavement performance requirements.

Overall, no single hybrid mixture provided the best performance for all evaluated properties. The results demonstrate that the mechanical response of hybrid-modified asphalt mixtures depends on the combined influence of diatomite type, diatomite content, and fibre type. Therefore, the selection of an optimum hybrid modification system should be based on the targeted pavement performance requirements and service conditions rather than on a single performance indicator.

## 5. Recommendations and Limitations

The present study was limited to a single aggregate gradation, a single binder grade, and one fibre dosage for each fibre type. In addition, mixtures containing only diatomite or only fibres were not included; therefore, the individual contributions of each modifier could not be isolated from the hybrid modification effect. Future studies should investigate different fibre dosages, include single-modifier mixtures for comparison, and evaluate the applicability of hybrid diatomite–fibre modification using different aggregate gradations and binder types, particularly in open-graded asphalt mixtures where the contribution of diatomite to abrasion resistance may be more pronounced.

Although the laboratory tests provided a comprehensive evaluation of the mechanical performance of the investigated hybrid-modified asphalt mixtures, validation under long-term pavement service conditions was beyond the scope of the present study. Therefore, future studies should validate the laboratory findings through field trials, accelerated pavement testing, or long-term pavement performance monitoring to assess the long-term durability and practical field applicability of the proposed hybrid modification system.

Although the proposed interpretation is consistent with the binder absorption characteristics of diatomite reported in previous studies [19,20,21,22,68], direct microstructural characterization (e.g., SEM or FTIR) and binder absorption measurements were beyond the scope of the present study. Therefore, future studies should incorporate these techniques to directly verify the proposed reinforcement and moisture-damage mechanisms.

Future research should also include comprehensive cost–performance, life-cycle cost, and environmental assessments to further evaluate the economic feasibility and practical implementation of hybrid diatomite–fibre-modified asphalt mixtures for large-scale pavement applications.

## Figures and Tables

**Table 1:** Properties of the base binder.

Test	Result	Specification Limits	Test Method
Specific gravity (25 °C)	1.025	1.0–1.1	ASTM D70 [35]
Softening point (°C)	50	46–54	TS EN 1427 [36]
Flash point (°C)	240	≥230	TS EN ISO 2592 [37]
Penetration (25 °C) 0.1 mm	51.30	50–70	TS EN 1426 [38]
Solubility (%)	99	≥99	TS EN 12592 [39]

**Table 3:** Physical and mechanical properties of PAN, PET, and basalt microfibres.

Microfibres	Length (mm)	Diameter (μm)	Density (g/cm^3^)	Tensile Stress (MPa)	Melting Point (°C)
PAN	6–12	13 ± 3	1.18	600–900	210
PET	6 ± 1.5	20 ± 5	1.36 ± 0.04	>700	260
Basalt	6–12	7–30	2.8	1050–3500	600

**Table 4:** Marshall Mix Design Parameters of Asphalt Mixtures with Different Binder Types.

Mix Design Parameters	Control	SBS%5	D1%10	D1%15	D2%10	D2%15	Specification Limit [34]
Marshall stability, kg	1796	1947	1839	1827	1865	1730	≥900
Voids filled with asphalt (VFA), %	74.81	74.91	75.08	74.82	75.14	74.86	65–75
Flow, mm	3.67	3.57	3.72	3.49	3.88	3.81	2–4
Voids in mineral aggregate (VMA), %	15.88	15.99	15.98	15.99	15.77	15.99	14–16
Filler/Bitumen Ratio	0.87	0.85	0.85	0.85	0.87	0.84	≤1.5
Optimum binder content (OBC), %	5.78	5.90	5.87	5.91	5.72	5.83	4–7

**Table 5:** Designation and Coding of Asphalt Mixtures Prepared in This Study.

Mixture Code	Modifier Type	Modifier Content (wt.%)	Microfibre Type
D1%10 PAN	D1	10	PAN
D1%15 PAN	D1	15	PAN
D1%10 PET	D1	10	PET
D1%15 PET	D1	15	PET
D1%10 BSLT	D1	10	Basalt
D1%15 BSLT	D1	15	Basalt
D2%10 PAN	D2	10	PAN
D2%15 PAN	D2	15	PAN
D2%10 PET	D2	10	PET
D2%15 PET	D2	15	PET
D2%10 BSLT	D2	10	Basalt
D2%15 BSLT	D2	15	Basalt
SBS%5	SBS	5	-
Control	-	-	-

**Table 6:** Creep slope, area between the creep curves, and standard deviation values of the control and modified asphalt mixtures.

Mixture	Area Between the Most Divergent Creep Curves (mm·Load Cycle)	Standard Deviation of Deformation at 10,000 Load Cycles (mm)
CONTROL	1967.1	0.1004
SBS%5	1889.5	0.097
D1%10 PAN	624.9	0.034
D1%10 PET	2194.4	0.112
D1%10 BSLT	1997.3	0.104
D2%15 PAN	2741.9	0.139
D2%15 PET	1563.8	0.084
D2%15 BSLT	1644.8	0.084
D2%10 PAN	1230.2	0.065
D2%10 PET	1476.7	0.077
D2%10 BSLT	1790.4	0.091
D1%15 PAN	1364.8	0.072
D1%15 PET	1672.2	0.087
D1%15 BSLT	1803.9	0.0934

**Table 7:** Summary of the factorial ANOVA results.

Performance Parameter	Diatomite Type	Diatomite Content	Fibre Type	Significant Interactions
ITS, 0 °C	<0.001 *	0.018 *	0.053	None
Permanent deformation	0.991	<0.001 *	0.464	None
Cantabro loss	<0.001 *	0.084	0.381	None
G_f_	0.001 *	<0.001 *	0.053	D × F, C × F, D × C × F
|m|	0.043 *	0.007 *	0.003 *	D × C, D × F, C × F
FI	<0.001 *	<0.001 *	<0.001 *	D × F, C × F, D × C × F

* Statistically significant (*p* < 0.05). D: diatomite type; C: diatomite content; F: fibre type.

## Data Availability

The original contributions presented in this study are included in the article. Further inquiries can be directed to the corresponding author.

## Appendix A

To improve the conciseness of the main manuscript, the supplementary aggregate characterization data that are not essential to the interpretation of the main findings have been transferred to this appendix. These data are provided for completeness and to facilitate reproducibility of the experimental work.

**Table A1:** Aggregate Properties.

Properties	Test Result	Test Standard
Filler apparent specific gravity	3.005	TS EN 1097-6 [40]
Fine aggregate specific gravity	2.833	TS EN 1097-6 [40]
Fine aggregate apparent specific gravity	2.967	TS EN 1097-6 [40]
Fine aggregate absorption (%)	1.59	TS EN 1097-6 [40]
Coarse aggregate specific gravity	2.848	TS EN 1097-6 [40]
Coarse aggregate apparent specific gravity	2.937	TS EN 1097-6 [40]
Coarse aggregate absorption (%)	1.05	TS EN 1097-6 [40]
Effective specific gravity of the mixture	2.905	ASTM D-2041 [41]
